# Transcriptome profiling of long noncoding RNAs and mRNAs in spinal cord of a rat model of paclitaxel-induced peripheral neuropathy identifies potential mechanisms mediating neuroinflammation and pain

**DOI:** 10.1186/s12974-021-02098-y

**Published:** 2021-02-18

**Authors:** Yuanyuan Li, Chengyu Yin, Boyu Liu, Huimin Nie, Jie Wang, Danyi Zeng, Ruixiang Chen, Xiaofen He, Junfan Fang, Junying Du, Yi Liang, Yongliang Jiang, Jianqiao Fang, Boyi Liu

**Affiliations:** Department of Neurobiology and Acupuncture Research, The Third Clinical Medical College, Zhejiang Chinese Medical University, Key Laboratory of Acupuncture and Neurology of Zhejiang Province, 548 Binwen Road, Hangzhou, 310053 China

**Keywords:** RNA-Seq, LncRNA, Paclitaxel, Neuropathic pain, Inflammation

## Abstract

**Background:**

Paclitaxel is a widely prescribed chemotherapy drug for treating solid tumors. However, paclitaxel-induced peripheral neuropathy (PIPN) is a common adverse effect during paclitaxel treatment, which results in sensory abnormalities and neuropathic pain among patients. Unfortunately, the mechanisms underlying PIPN still remain poorly understood. Long noncoding RNAs (lncRNAs) are novel and promising targets for chronic pain treatment, but their involvement in PIPN still remains unexplored.

**Methods:**

We established a rat PIPN model by repetitive paclitaxel application. Immunostaining, RNA sequencing (RNA-Seq) and bioinformatics analysis were performed to study glia cell activation and explore lncRNA/mRNA expression profiles in spinal cord dorsal horn (SCDH) of PIPN model rats. qPCR and protein assay were used for further validation.

**Results:**

PIPN model rats developed long-lasting mechanical and thermal pain hypersensitivities in hind paws, accompanied with astrocyte and microglia activation in SCDH. RNA-Seq identified a total of 814 differentially expressed mRNAs (DEmRNA) (including 467 upregulated and 347 downregulated) and 412 DElncRNAs (including 145 upregulated and 267 downregulated) in SCDH of PIPN model rats *vs*. control rats. Functional analysis of DEmRNAs and DElncRNAs identified that the most significantly enriched pathways include immune/inflammatory responses and neurotrophin signaling pathways, which are all important mechanisms mediating neuroinflammation, central sensitization, and chronic pain. We further compared our dataset with other published datasets of neuropathic pain and identified a core set of immune response-related genes extensively involved in PIPN and other neuropathic pain conditions. Lastly, a competing RNA network analysis of DElncRNAs and DEmRNAs was performed to identify potential regulatory networks of lncRNAs on mRNA through miRNA sponging.

**Conclusions:**

Our study provided the transcriptome profiling of DElncRNAs and DEmRNAs and uncovered immune and inflammatory responses were predominant biological events in SCDH of the rat PIPN model. Thus, our study may help to identify promising genes or signaling pathways for PIPN therapeutics.

**Supplementary Information:**

The online version contains supplementary material available at 10.1186/s12974-021-02098-y.

## Introduction

Chemotherapy-induced peripheral neuropathy (CIPN) is a dose-limiting neurotoxic adverse effect of many chemotherapeutic agents [1]. Paclitaxel is one of the most commonly used chemotherapeutic reagents to treat breast, ovarian, non-small cell lung cancer, etc. [2, 3]. Paclitaxel-induced peripheral neuropathy (PIPN) is a common and serious side effect accompanying paclitaxel treatment [1]. Neuropathic pain is a major clinical symptom of PIPN, which includes tingling, burning pain, and numbness in feet and hands [4, 5]. The neuropathic pain symptoms of PIPN occur among 50–100% of patients receiving chemotherapy, depending on the doses [6, 7]. The painful peripheral neuropathy is the most dose-limiting side effect of taxanes [8]. The sensory abnormalities and pain can even become chronic and persist after paclitaxel treatment is terminated, which severely affect the life quality of the patients [9]. Conventional treatments for paclitaxel-induced peripheral neuropathic pain include nonsteroidal anti-inflammatory drugs, opioids, corticosteroids, and antidepressants [10]. However, these medical treatments are usually insufficient for paclitaxel-induced peripheral neuropathic pain management and oftentimes resulted in a number of severe side effects [10]. Therefore, paclitaxel-induced peripheral neuropathic pain still remains a challenging clinical problem for patients receiving chemotherapy.

Emerging evidence suggests an important role of glial cells (such as astrocytes and microglia) in spinal cord dorsal horn (SCDH) in mediating CIPN. SCDH receives pain signal inputs from the peripheral sensory neurons and plays a pivotal role in integrating pain signals and central pain sensitization. We and others have found that astrocytes and microglia are activated in SCDH of PIPN model rats [11–13]. The activation of these cells produces several pro-inflammatory cytokines or chemokines, such as IL-1β, IL-17, TNF-α, and CXCL-12, which initiate neuroinflammation and regulate neuronal excitability and contribute to pain mechanisms of PIPN [12–14].

Long noncoding RNAs (lncRNAs) are a class of non-coding RNAs with sequence length greater than 200 nucleotides yet without protein coding potential [15, 16]. LncRNAs can interact with proteins, DNAs, and other RNAs and get involved in transcriptional modulation or regulating multiprotein complexes [17]. There are growing number of studies reporting that lncRNA expression can be modulated by neuronal activity and injury [18, 19]. Recently, emerging evidence suggested that dysregulated expression of lncRNA (DElncRNA) occurred in damaged nerves, dorsal root ganglions (DRGs), and SCDH, following peripheral nerve injury [20–22]. These DElncRNAs contribute to chronic pain mechanisms via modulating pain-related gene expression, such as P2X3 and KCNA2, in the peripheral and central sensory system [17, 20, 21, 23]. Although the studies of lncRNA’s contribution to chronic pain have attracted more and more attention, the role of lncRNA in mediating paclitaxel-induced peripheral neuropathic pain still remained elusive.

To further explore the central mechanisms underlying paclitaxel-induced peripheral neuropathic pain, in this study, we performed a genome-wide RNA-Seq of SCDH of a rat model of PIPN to explore expression profile changes of lncRNAs and mRNAs. We further investigated the major pathways or functions that these DEGs are involved in. Our study may provide insights into understanding the central mechanisms of PIPN and help to find promising genes or signaling pathways that can be targeted for PIPN therapy.

## Materials and methods

### Animals

Male Sprague–Dawley rats (5–8 weeks, 180–220 g) were purchased from Shanghai Laboratory Animal Center, Chinese Academy of Sciences and housed in the Laboratory Animal Center of Zhejiang Chinese Medical University accredited by the Association for Assessment and Accreditation of Laboratory Animal Care (AAALAC). The rats were randomly allocated and were housed in a controlled environment (5 rats per cage on 12 h light–dark cycles with controlled temperature). Food and water were provided ad libitum. The rats were given a minimum of 1 week to adapt to new environment before the experiment. All experimental procedures were approved by the Animal Ethics Committee of Zhejiang Chinese Medical University (ZSLL-2017-183).

### Model establishment

The rat PIPN model was established according to methods previously described [11, 24, 25]. Briefly, 6 mg/mL of pharmaceutical grade paclitaxel (Hospira Australia Pty. Ltd., Australia) was diluted with sterile 0.9% saline to 1 mg/mL and given at a dosage of 2 mg/kg intraperitoneally (i.p.) in a volume of 0.5 ml/250 g rat every other day for a total of four injections (days 1, 3, 5, and 7). Control animals received the same volume of sterile saline treatment.

For the study to investigate the vehicle effect of paclitaxel formulation, rats received an equivalent volume of the vehicle for paclitaxel, with proportional amounts of Cremophor EL (C875008, Macklin Inc., Shanghai, China) and 95% dehydrated ethanol diluted in sterile 0.9% saline (Cremophor EL/ethanol 1:1). Control rats received equal amount of saline injection (0.5 ml/250 g). Rats were observed carefully for any abnormal behavioral changes every day following the treatment.

### Mechanical allodynia

Rats were placed in the test environment daily for 3 consecutive days before baseline test to habituate the test environment. Before the test, rats were individually placed in transparent Plexiglas chambers on an elevated mesh floor to acclimate for 30 min. The mechanical allodynia was determined using a series of von Frey filaments (UGO Basile, Italy) applied perpendicularly to the mid-plantar surface of the hind paws, with sufficient force to bend the filament slightly for 3–5 s according to methods we previously used [26, 27]. An abrupt withdrawal of the hind paw, licking or vigorously shaking in response to stimulation was considered as pain-like responses. The threshold was determined using the up-down testing paradigm and the 50% paw withdrawal threshold (PWT) was calculated by the nonparametric Dixon test [28, 29]. A baseline test of PWT was done every day for 3 consecutive days before formal testing to acclimatize the rats and to ensure that there were no differences among groups.

### Thermal hyperalgesia

The Plantar Test Apparatus (Ugo Basile, Italy) was used to evaluate thermal hyperalgesia as described before [30]. Rats were habituated for 30 min before the test. A radiant light beam generated by a light bulb was directed into the right hind paw in order to determine the paw withdrawal latency (the time spent to remove the paw from the stimulus). A 20-s cutoff threshold was set to avoid excessive heating to cause injury. Significant decreases in paw withdrawal latency (PWL) were interpreted as heat hyperalgesia. All behavior tests were performed by an experimenter blinded to groupings.

### Tissue collection and RNA extraction

At day 14, rats were deeply anesthetized with sodium pentobarbital (40 mg/kg, i.p.) and perfused through the ascending aorta with 0.9% saline (4 °C). After the perfusion, the spinal cord dorsal horn segments were collected. Total RNA was extracted from the control and Pac group tissues using Trizol reagent (Invitrogen, Carlsbad, CA, USA), following the manufacturer’s instructions. For tissue samples, about 60 mg with liquid nitrogen was ground into powder and the powder samples were transferred into the 2-ml tube which contains 1.5 ml Trizol reagent. The mixture was centrifuged at 12,000×*g* for 5 min at 4 °C. The supernatant was transferred to a new 2.0-ml tube with 0.3 ml of chloroform/isoamyl alcohol (24:1) per 1.5 ml of Trizol reagent. After the mixture was centrifuged at 12,000×*g* for 10 min at 4 °C, the aqueous phase was transferred to a new 1.5 mL tube which was added with an equal volume of supernatant of isopropyl alcohol. The mixture was centrifuged at 12,000×*g* for 20 min at 4 °C and then the supernatant was removed. After being washed with 1 ml 75% ethanol, the RNA pellet was air-dried in the biosafety cabinet and then dissolved by adding 25 ~ 100 μL DEPC-treated water. Subsequently, total RNA was qualified and quantified using a Nano Drop and Agilent 2100 Bioanalyzer (Thermo Fisher Scientific, MA, USA).

### RNA-Seq library establishment and RNA-Seq

Approximately 1 μg total RNA per sample was treated with Ribo-Zero™ Magnetic Kit (Epicentre) to deplete rRNA. First-strand cDNA was generated using random primers reverse transcription, followed by a second-strand cDNA synthesis. The synthesized cDNA was subjected to end-repair and then was 3′ adenylated. Adapters were ligated to the ends of these 3′ adenylated cDNA fragments. Several rounds of PCR amplification with PCR Primer Cocktail and PCR Master Mix are performed to enrich the cDNA fragments. Then the PCR products are purified with Ampure XP Beads. The final library was quality and quantitated in two methods: checking the distribution of the fragment size using the Agilent 2100 Bioanalyzer and quantifying the library using real-time quantitative PCR (qPCR) (TaqMan Probe). The qualified libraries were sequenced pair end on the Hiseq 4000 platform (BGI-Shenzhen, China).

### Bioinformatics analysis

Primary sequencing data produced by RNA-Seq (raw reads) were subjected to quality control (QC). The information of total reads and mapping ratio reads are shown in Table 1. The sequencing data was filtered with SOAPnuke (v1.5.2) [31] by removing reads containing sequencing adapter; removing reads whose low-quality base ratio (base quality less than or equal to 5) is more than 20%; and removing reads whose unknown base (“N” base) ratio is more than 5%; afterwards, clean reads were obtained and stored in FASTQ format. The clean reads were mapped to the reference genome using HISAT2 (v2.0.4) [32]. After that, Ericscript (v0.5.5) [33] and rMATS (V3.2.5) [34] were used to detect fusion genes and differential splicing genes (DSGs), respectively. Bowtie2 (v2.2.5) was applied to align the clean reads to the gene set [35], a database built by BGI (Beijing Genomic Institute in Shenzhen), in which known and novel, coding and noncoding transcripts were included, then expression level of gene was calculated by RSEM (v1.2.12) [36]. The heatmap was drawn by pheatmap (v1.0.8) according to the gene expression in different samples. Essentially, differential expression analysis was performed using the DESeq2(v1.4.5) with *q* value ≤ 0.05 [37]. To take insight into the change of phenotype, GO (http://www.geneontology.org/) and KEGG (https://www.kegg.jp/) enrichment analysis of annotated different expression gene was performed by Phyper (https://en.wikipedia.org/wiki/Hypergeometric_distribution) based on the hypergeometric test. The significant levels of terms and pathways were corrected by *q* value with a rigorous threshold (*q* value ≤0.05) by Bonferroni.

**Table 1:** The information of total reads and mapping ratio for control and PIPN groups by RNA-Seq

Sample	Total raw reads (Mb)	Total clean reads (Mb)	Clean reads Q30 (%)	Clean reads ratio (%)	Total mapping ratio (%)
Con1	137.1	126.2	96.80	92.052	83.34
Con2	126.1	115.2	96.97	91.367	84.15
Con3	137.2	125.9	96.99	91.723	85.08
Con4	136.8	126.1	97.08	92.116	83.73
Pac 1	138.7	126.2	97.07	90.924	85.55
Pac 2	138.7	126.7	97.02	91.274	86.60
Pac 3	138.7	127.0	97.07	91.512	86.63
Pac 4	138.7	127.2	96.99	91.638	86.27

### Cluster analysis and screening of differentially expressed genes

Distances of expressed genes were calculated using the Euclidean method [38]. The sum of the squared deviations algorithm was used to calculate distance. The cluster analysis and heat map visualization of gene expression patterns were performed using the “pheatmap” package in the R software of Bioconductor. Differentially expressed mRNAs with statistical significance were identified through Scatter Plot filtering as we reported before [39]. The threshold required for the results to be considered significant was as follows: *q* value ≤ 0.01 and absolute value of |log_2_ (fold change) | ≥ 1.0 as in our previous study [26].

### Functional enrichment analysis of DEGs

The differentially expressed mRNAs were selected and subjected to gene ontology (GO) and Kyoto Encyclopedia of Genes and Genomes (KEGG) pathway analysis. For GO analysis (http://geneontology.org/), the corresponding genes were annotated and classified according to biological process (BP), cellular component (CC), and molecular function (MF). For KEGG analysis (http://www.genome.jp/kegg/), pathways were ranked by their enrichment scores. Cluster analysis and screening of differentially expressed genes.

### Real-time quantitative PCR (qPCR)

The extracted total RNA from the spinal cord was reverse-transcribed into cDNA using random hexamer primers (TaKaRa Bio Inc., Shiga, Japan) according to the manufacturer’s instruction. The sequences of all primers used are shown in Table 2. qPCR was performed using spinal cord tissues from rats other than the ones used for RNA-Seq. β-actin was used as an internal reference gene. qPCR was performed using the Fast Start Universal SYBR Green Master kit (TaKaRa Bio Inc., China) with 25 μL reaction system according to the manufacturer’s protocol by CFX96 Real-Time System (Bio-Rad Laboratories Inc., Hercules, CA, USA). Each reaction was performed in triplicates and normalized to β-actin gene expression. The CT value of each well was determined using the CFX96 Real-Time System software and the average of the triplicates was calculated. The relative quantification was determined by the ΔΔCT method [40, 41].

**Table 2:** Sequences of the primers used for qPCR validation of RNA-Seq data

Gene name	Primer sequence (5′ to 3′)	Amplicon size (bp)
Beta-actin	Forward: TGTCACCAACTGGGACGATA	165
	Reverse: GGGGTGTTGAAGGTCTCAAA	
Cxcl13	Forward: CGACTTTGAAAGGTTGCTTGTA	219
	Reverse: ACACTGGATGAATAGGAAACGT	
Ccl3	Forward: AGAGGCAGCGAGTACCAGTCC	184
	Reverse: CATAGGAGAAGCAGCAGGCAGTC	
Cxcl11	Forward: GCTGTGACAAAGTTGAAGTGAT	102
	Reverse: GTATTGTCTGCATTATGAGGCG	
Cd68	Forward: GCTGTGACAAAGTTGAAGTGAT	200
	Reverse: GGCTGGTAGGTTGATTGTCGTCTC	
Mettl14	Forward: TGAGAGGCTTAGACCGAAGTCACC	104
	Reverse: GACCACGGCCAGCAGATGTTC	
Pcdh18	Forward: CCAGAGTCACAACAGTCACCAGTC	109
	Reverse: CTCAACAGCAGGAGTGGCATGG	
Nap1l4	Forward: GGCACAGTGAGAATGAGGAGGATG	163
	Reverse: ACCAGTTCGCTAAGCATGTCCAC	
LOC691141	Forward: AGGCGGTCCAGCTCCTACAAG	121
	Reverse: ACACCATACTGACAGCGGAAGTTG	
Pdk4	Forward: GGCCACGGTCGAGCATCAAG	168
	Reverse: GGCGTTGGAGCAGTGGAGTATG	
Slco2a1	Forward: ATCCTATTCGCCATCGCTGTGTTC	94
	Reverse: TGTCCACTCTGCCGTAGTCCAC	
Fgf21	Forward: TCTCCTGCTGCCTGTCTTCCTG	129
	Reverse: TCGGTGTCCTGGTCGTCATCTG	
Zbtb16	Forward: CACCAGCAAGATGTTTGAGATC	239
	Reverse: CTGTGTCATTGTCATCAGATGC	
Slc10a6	Forward: TCCTTCTCTGCTGAGTACCTGGTC	102
	Reverse: TGCCTGATATGCTGCGACAATGAG	
102552154	Forward: ATCGCTAGTTATGCAGGCATGGAG	124
	Reverse: CAGCCAATGAGTTGCACTTGAACC	
108351753	Forward: CAGTGGTTGTGAGCTGTCTGAGG	148
	Reverse: ACGCAAGCATGGTAGGTGTTGG	
102546487	Forward: AGCCTCAGCCTTAAGCATGTGTTC	85
	Reverse: CCTTGTCTCCTCGGTTGGATGTG	
108349645	Forward: AGGAGCACGGAGATAGCAACTGG	108
	Reverse: CGGTGAGTACAGTGCTTGCCATAC	
108352578	Forward: CGCGAACACCGGAACTCTTAGATC	125
	Reverse: TGCCACTTGTGCTCAGCTTCTTC	

### Immunofluorescence staining

Rats were sacrificed on day 14. Rats were deeply anesthetized with sodium pentobarbital (40 mg/kg, i.p.) and perfused through the ascending aorta with 0.9% saline (4 °C) followed by 4% paraformaldehyde in 0.1 M PBS. The L4–6 dorsal root ganglia (DRGs) and the spinal cord were removed, fixed in 4% paraformaldehyde for 6 h, and then cryo-protected in 30% sucrose solution. Transverse spinal cord sections (25 μm) and longitudinal DRG sections (10 μm) were cut on a frozen microtome (Thermo NX50, MA, USA), mounted on gelatin-coated glass slides as 8 sets of every 5th serial section, and processed for immunofluorescent staining. After blocking in 5% normal donkey serum in Tris-buffered saline tween (TBST) for 1 h at 37 °C, they were incubated overnight with corresponding primary antibodies. The primary antibodies used were rabbit anti-ATF3 (HPA001562, 1:400, Sigma, USA), mouse anti-GFAP (c9205, 1:400, Sigma, USA), mouse anti-OX42 (ab1211, 1:600, Abcam, UK), and mouse anti-NeuN (ab104224, 1:500, Abcam, UK). After washing, the sections were then incubated with Cy3-, Cy5-, or fluorescein isothiocyanate (FITC)-conjugated secondary antibodies (Abcam, UK) for 1 h at 37 °C. Sections were viewed by a Nikon A1R laser scanning confocal microscope (Nikon, Japan). For image quantification, uniform microscope settings were maintained throughout all image capture sessions and experimenters were blinded to treatment groups. Three to five images were randomly selected per rat tissue, averaged, and then compared according to methods described in our previous studies [11, 30].

### Western blot

Rats were sacrificed after behavioral test on day 14. Rats were deeply anesthetized with sodium pentobarbital (Nembutal, 40 mg/kg, i.p.) and perfused through the ascending aorta with 0.9% saline (4 °C). Then, the SCDH were rapidly removed on ice. Tissues were immediately removed and stored at − 80 °C. Tissues were homogenized in radioimmunoprecipitation assay (RIPA) buffer (50 mM Tris (pH 7.4), 150 mM NaCl, 1% Triton X-100, 1% sodium deoxycholate, sodium orthovanadate, 0.1% sodium dodecyl sulfonate (SDS), ethylenediamine tetraacetic acid (EDTA), sodium fluoride, leupeptin, and 1 nM PMSF). The homogenate was allowed to rest on ice for 30 min and was then centrifuged at 15,000 rpm for 15 min at 4 °C, and the supernatant was collected. The protein concentration was determined using the bicinchoninic acid (BCA) method according to the kit’s instruction (Thermo Fisher, USA), and 15 μg of protein was loaded in each lane. Protein samples were separated on 5–12% SDS-PAGE gels and electrophoretically transferred to polyvinyl difluoride (PVDF) membranes (Bio-Rad, USA). The membranes were blocked with 5% non-fat milk in Tris-buffered saline (TBS) with 0.1% Tween-20 (pH 7.5) at room temperature for 1 h, followed by overnight incubation at 4 °C with the following primary antibodies diluted in blocking buffer: mouse anti-CXCL11 (ab9955, 1:1000, Abcam, UK). Subsequently, the immunoblots were incubated with the 2nd antibodies for 2 h at room temperature. Mouse anti-beta-actin-loading control (HRP) (ab20272, Abcam, UK) was used as internal control. The immunoreactivity was detected using enhanced chemiluminescence (BIO-RAD, USA) and visualized with an Image Quant LAS 4000 (GE, USA). The density of each band was measured using Image Quant TL 7.0 analysis software (GE, USA). The mean expression level of the target protein in the animals in the vehicle group was considered as 100%, and the relative expression level of the target protein in all animals was adjusted as a ratio to the level of the vehicle group.

### ELISA

The rat SCDH tissues were collected and homogenized as described above in Western blot section. The homogenate was allowed to rest on ice for 30 min and was then centrifuged at 15,000 rpm for 15 min at 4 °C, and the supernatant was collected. The supernatant was used for ELISA testing of CCL3 (Abcam, UK) according to the manufacturer’s instruction.

### Source of microarray data

Two independent datasets of neuropathic pain models were selected for the study: spared nerve injury (SNI) and chronic constriction injury (CCI) neuropathic pain model microarray. The SNI dataset (GSE18803) was downloaded from Gene Expression Omnbius (GEO), at the website of https://www.ncbi.nlm.nih.gov/geo/. The CCI dataset comes from a recently published article on CCI sequencing [28]. Differentially expressed genes from these microarray datasets were screened based on criteria as *q* value ≤ 0.01 and absolute value of |log_2_ (fold change) | ≥ 1.0.

### Protein-protein interaction (PPI) network analysis

The Search Tool for the Retrieval of Interacting Genes (STRING) is used to provide information regarding predicted and experimental interactions of proteins and the prediction method of this database is from neighborhood, gene fusion, co-occurrence, co-expression experiments, databases, and text mining. By setting the combination score > 0.4 as the reliability threshold value, the web-based STRING database (http://string-db.org/) was used to produce PPI predictions after uploading the union gene list to the search bar [42]. Based on the interplayed relationships, a PPI network was established and then visualized using the Cytoscape software [43]. The connectivity degree of each protein, namely the number of proteins it connected, was calculated to evaluate its importance in this network.

### Competing endogenous RNA (ceRNA) analysis of DElncRNAs and DEmRNAs

The possible target binding of lncRNA/mRNA and miRNA was predicted by Dr. Tom system from BGI with database TargetScan, miRanda and RNAhybrid. Based on the interplayed relationships, a network was established and then visualized using the Cytoscape software [43].

### Statistical analysis

Statistical analysis was performed with SPSS 19.0 (IBM Corp., Armonk, NY, USA). One- or two-way ANOVA followed by Tukey’s post hoc test was used for comparison among groups ≥ 3. Student’s *t* test was used for comparisons between two groups. Data in graphs are expressed as means ± SEM. Comparison is considered significantly different if *p* < 0.05.

## Results

### Establishment of the rat model of PIPN

**Fig. 1:**
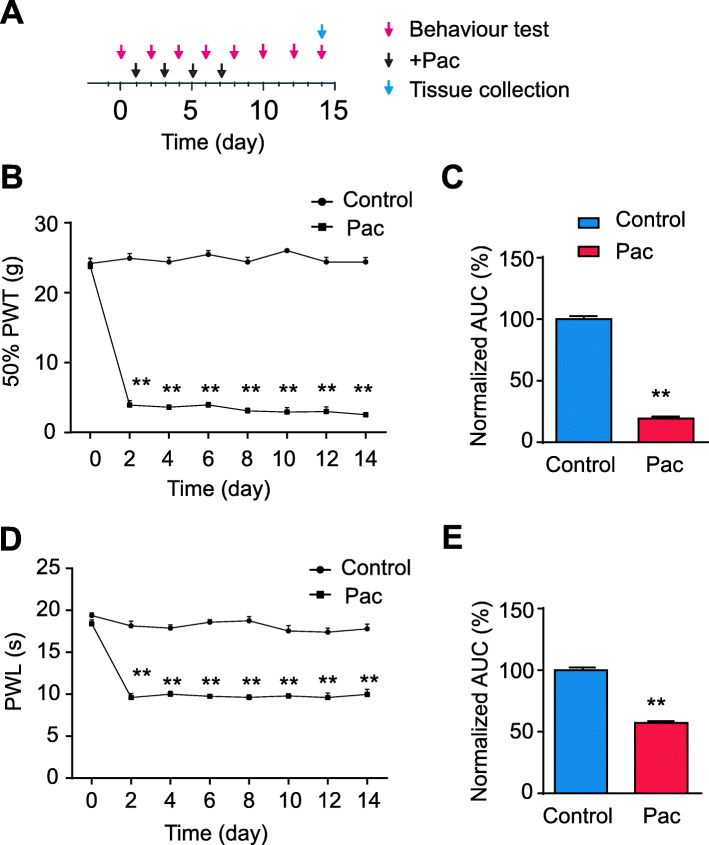
The rat model of PIPN exhibits persistent mechanical and thermal hypersensitivities. **a** Experimental protocol for the establishment of the rat model of paclitaxel-induced peripheral neuropathy (PIPN). **b** 50% paw withdraw threshold (PWT) of right hind paw of control and Pac group rats. **c** Normalized AUC analysis of panel (**b**). **d** 50% paw withdraw latency (PWL) of right hind paw of control and Pac group rats. **e** Normalized AUC analysis of panel (**d**). AUCs were all normalized to corresponding control group. ^**^p < 0.01 *vs*. control group. *n* = 5 rats/group. Two-way ANOVA followed by Tukey post hoc test was used for statistical analysis in panels (**b** and **d**), whereas Student’s *t* test was used for statistical analysis in panels (**c** and **e**)

We first established the rat model of PIPN via multiple paclitaxel injections (Fig. 1a). Administration of a cumulative dosage of 8 mg/kg paclitaxel (4 × 2 mg/kg, 2 days apart, intraperitoneal (i.p.) injection) elicited a robust and persistent reduction in 50% paw withdraw threshold (PWT) in paclitaxel-treated group rats (Pac group) (Fig. 1b), compared with vehicle-treated rats (control group). The mechanical allodynia lasted till the end of our observation time frame (day 14). In addition, Pac group rats also developed obvious signs of thermal hyperalgesia, manifested by a significant reduction of paw withdraw latency (PWL) by Hargreaves test, as compared with control group. The thermal hyperalgesia persisted until the end of the observation time frame as well (day 14) (Fig. 1d). These results were consistent with previous reports, indicating successful model establishment of PIPN model in rats.

### PIPN model rats showed over-activation of astrocytes and microglia but no neuronal damage in SCDH

**Fig. 2:**
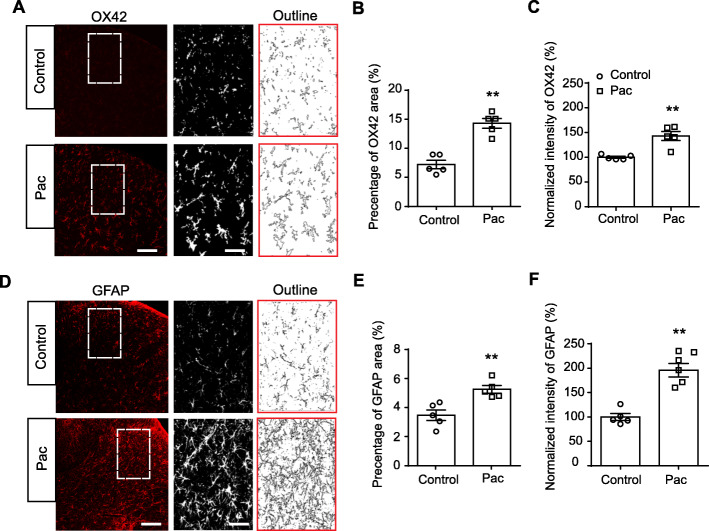
Overexpression of OX42 and GFAP in SCDH of paclitaxel-treated rats. **a** Representative immunofluorescence images indicating OX42 (a microglia marker) antibody staining of spinal cord from control and paclitaxel-treated (Pac) groups. Areas staining positive for ATF3 are shown in red. Each dorsal horn was outlined for further analysis. Microglia were further magnified in SCDH and outlines of microglia were illustrated in the right panel. Scale bars indicates 100 μm (entire spinal dorsal horn) and 25 μm (magnified dorsal horn), respectively. **b** Percentage of OX42 positively staining area in each observation field. **c** Summary of the normalized % increase in fluorescence intensity of OX42 immunostaining in each observation field. The value of each group was normalized to that of control group. **d** Representative immunofluorescence images indicating GFAP (an astrocyte marker) antibody staining of spinal cord from control and Pac groups. Areas staining positive for GFAP are shown in red. Astrocytes were further magnified and outlines of astrocytes were illustrated in the right panel. Scale bars indicates 100 μm (entire spinal dorsal horn) and 25 μm (magnified dorsal horn), respectively. **e** Percentage of GFAP positively staining area in each observation field. **f** Summary of the normalized % increase in fluorescence intensity of GFAP immunostaining in each observation field. The value of each group was normalized to that of control group. *n* = 5 rats/group. ^**^p < 0.01 vs. control group. Student’s *t* test was used for statistical analysis in panels (**b**, **c**, **e**, and **f**)

Astrocytes and microglia in spinal cord dorsal (SCDH) horn play important roles in mediating chronic pain. Our recent work, together with others, found that astrocytes and microglia were over-activated in SCDH of PIPN model rats and involved in pain mechanisms [11–13, 44]. We confirmed these findings by performing immunostaining of SCDH using GFAP and OX42, markers for astrocyte and microglia, respectively. We found that the expressions of GFAP and OX42 were significantly increased in SCDH of PIPN model rats (Fig. 2a–f). This result indicated that astrocytes and microglia were over-activated in SCDH of PIPN model rats.

**Fig. 3:**
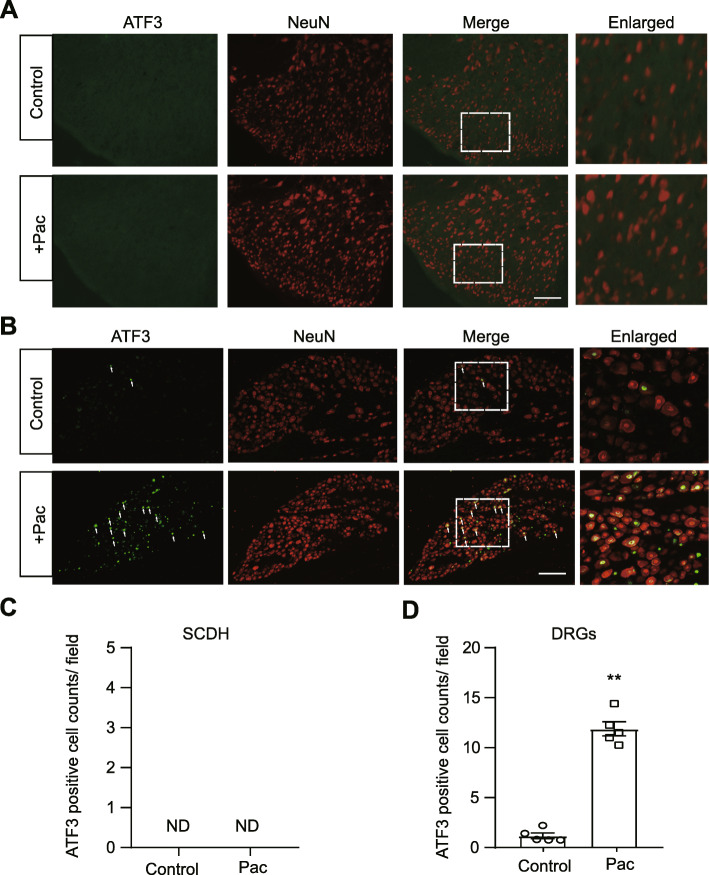
Neuronal damage examination of SCDH and DRGs of paclitaxel-treated rats. **a b** Representative immunofluorescence images indicating ATF3 antibody staining of SCDH (**a**) and DRGs (**b**) from control and paclitaxel-treated (Pac) groups. Areas staining positive for ATF3 are shown in green. NeuN (red) was used for neuron labeling. **c**, **d** Summary of ATF3 positively stained neurons per observation field in SCDH (**c**) and DRGs (**d**) from control and paclitaxel-treated groups. ND: not detectable. ^**^p < 0.01 *vs*. control group. *n* = 5 rats/group. Student’s *t* test was used for statistical analysis

We further evaluated whether neuronal damage occurred in SCDH after paclitaxel treatment. We used ATF3, a widely used marker for neuronal damage for the examination. As shown in Fig. 3a, c, we did not observe any ATF3 positively stained cells in spinal cord dorsal horn in paclitaxel-treated rats. As a positive control, we also stained peripheral DRGs with ATF3 and found that the number of ATF3 positively expressed neurons was significantly increased in DRGs of paclitaxel-treated rats *vs*. control rats (Fig. 3b, d), a result consistent with other studies showing that neuronal damage occurred in peripheral sensory neurons after paclitaxel treatment [45]. Therefore, this result showed that spinal cord neurons are not damaged after paclitaxel treatment. This result ruled out the possible contribution of neuronal damage in spinal cord to paclitaxel-induced peripheral neuropathic pain.

### Transcriptome profiling of SCDH of PIPN model rats by RNA-Seq

To gain insights into the mechanisms of paclitaxel-induced peripheral neuropathic pain, we harvested L4-L6 segment of SCDH of PIPN model rats and control rats and profiled the mRNA and lncRNA expression patterns using RNA-Seq technique. The sequencing produced approximate 1.092 billion raw reads per sample and the clean reads ratio was nearly 92.0% (Table 1). More than 96% of bases had a quality score ≥ Q30 and over 92% of the clean reads data were mapped to the rat genome (Table 1). A total of 20,796 mRNAs and 13,787 lncRNAs were successfully mapped and identified from RNA-Seq (Additional file 1: Suppl. Table 1)

**Fig. 4:**
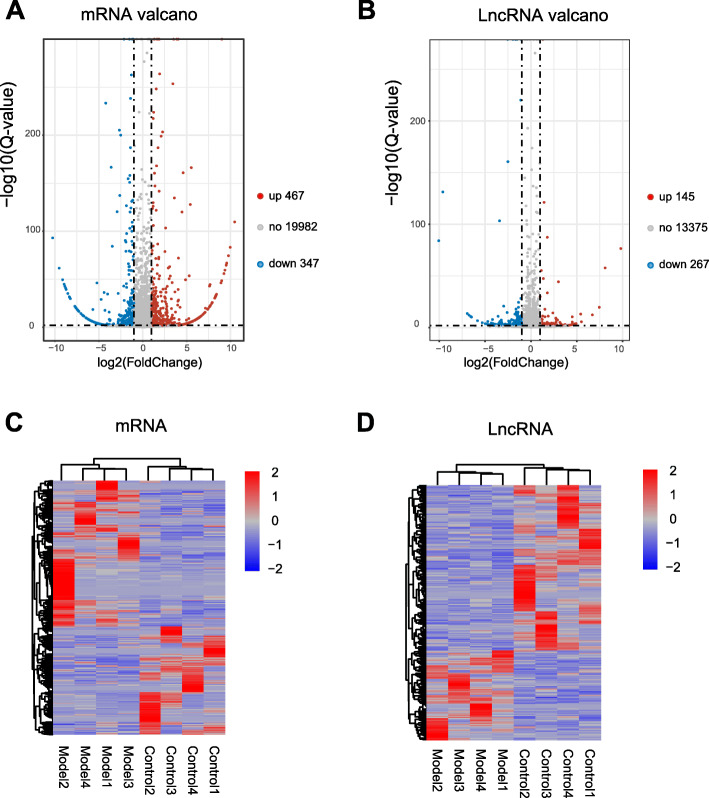
RNA-Seq identifies expression changes of mRNAs and lncRNAs in SCDH of PIPN model rats. **a**, **b** Volcano plots of DEmRNAs (**a**) and DElncRNAs (**b**) in SCDH of Pac group rats vs. control group rats. Red points refer to upregulated DEGs and blue points refer to downregulated DEGs, whereas grey spots indicate non-DEGs. **c**, **d** Heat map illustrations of hierarchical clustering analysis of differently expressed mRNA (DEmRNA) (**c**) and lncRNA (DElncRNA) (**d**) in SCDH from control and Pac group rats

We then filtered out the differentially expressed genes (DEGs) with a criterion of fold change ≥ 2 and *q* value ≤ 0.01. These DEGs are illustrated in a volcano plot (Fig. 4a, b). A total of 814 DEmRNAs (including 467 upregulated and 347 downregulated) were identified. In addition, a total of 412 DElncRNAs (including 145 upregulated and 267 downregulated) were identified as well (Fig. 4a, b, Additional file 2: Suppl. Table 2). The DEmRNA and DElncRNA were further summarized and laid out in a heat map (Fig. 4c, d). Hierarchical clustering analysis was then performed for further analysis. Results indicated that a clear segregation existed between Pac and control group, but no segregations existed within groups (Fig. 4c, d).

### Analysis of DEmRNAs and DElncRNAs in SCDH of PIPN model rats

Among the DEmRNAs and DElncRNAs we have obtained, some genes were well established to be involved in pain processes. These genes include as follows: *Ccl3* (C-C motif chemokine ligand 3, fold change = 15.22), *Cxcl11* (C-X-C motif chemokine 11 precursor, fold change = 3.9), *Cxcl13* (C-X-C motif chemokine 13 precursor, fold change = 12.37), *Cd68* (cd68 molecule, fold change = 2.3). We further analyzed the data and found that 109 DEmRNAs showed expression changes more than 30-fold, with 65 upregulated genes and 44 downregulated genes, such as *Kif5b* (kinesin family member 5B, fold change = 95.32), *Ccdc50* (coiled-coil domain containing 50, fold change = 34.96), *Pnpo* (pyridoxamine 5'-phosphate oxidase, fold change = -110.88), *Plk4* (polo-like kinase 4, fold change = -38.95). In total, 17 DElncRNA showed expression changes more than 30-fold, with 12 upregulated genes and 5 downregulated genes, such as *LOC 108353231* (fold change = 96.2), *LOC103693147* (fold change = 55.69), *LXLOC_012126* (fold change = -101.44), and *LXLOC_012123* (fold change = − 48.37). Tables 3, 4, 5, and 6 illustrate the detailed information of the top 20 up- and 20 downregulated DEmRNAs and DElncRNAs, respectively.

**Table 3:** The detailed information of the top 20-upregulated DEmRNA

Upregulated gene name	Gene ID	Location	***Q*** value	Log_**2**_ fold change (Pac/control)
*Mboat7*	308309	**1:NC_005100.4**	1.23E − 112	10.47045683
*Marchf2*	362849	**7:NC_005106.4**	5.09E−86	9.963392968
*Kif5b*	117550	**17:NC_005116.4**	2.97E−77	9.763083788
	LOC000654		1.80E−69	9.566262667
*Fam168b*	690188	**9:NC_005108.4**	2.90E−67	9.506567641
*Zzz3*	310958	**2: NC_005101.4**	4.48E−62	9.3586638
	MXLOC_008222		1.78E−58	9.248934774
*Mpz*	24564	**13:NC_005112.4**	0	9.003982058
*Lamb2*	25473	**8:NC_005107.4**	5.72E−51	8.997842807
*Ap3m1*	171126	**15:NC_005114.4**	9.07E−49	8.917523643
*RT1-Cl*	24977	**NW_001088046.1**	1.09E−36	8.403453182
	MXLOC_031701		1.57E−35	8.345896503
	MXLOC_002749		4.65E−35	8.321992109
*Cnmd*	81512	**15:NC_005114.4**	2.74E−34	8.282249284
*Aff4*	303132	**10:NC_005109.4**	3.14E−34	8.279159906
*Sptbn1*	305614	**14: NC_005113.4**	1.42E−33	8.244642313
*Abca1*	313210	**5: NC_005104.4**	1.35E−32	8.191829602
*Myof*	309499	**1:NC_005100.4**	4.81E−28	7.924093716
*LOC689986*	689986	**14:NC_005113.4**	5.07E−27	7.858189936
*Pacsin1*	29704	**20: NC_005119.4**	7.39E−25	7.710583606

**Table 4:** The detailed information of the top 20 downregulated DEmRNA

Downregulated gene name	Gene ID	Location	***Q*** value	Log_**2**_ fold change (Pac/control)
*Pnpo*	64533	10:NC_005109.4	6.19E−96	−10.25972871
	MXLOC_011484		3.47E−64	−9.509624869
*Cul4a*	361181	16:NC_005115.4	4.64E−52	−9.124415381
*Tmem216*	361727	1:NC_005100.4	2.33E−47	−8.951600521
*Helz*	287773	10:NC_005109.4	3.81E−45	−8.864420971
*Oas1i*	304507	12:NC_005111.4	3.27E−43	− 8.784895643
*Tns1*	301509	9:C_005108.4	8.70E−42	−8.724083939
*Myorg*	366360	5:NC_005104.4	1.44E−37	−8.531715478
*Stx1b*	24923	1:NC_005100.4	4.38E−34	−8.356525666
*Tmem151a*	309158	1:NC_005100.4	2.57E−33	−8.315606053
*Klhl20*	304920	1:NC_005112.4	4.81E−32	-8.24578303
	MXLOC_026819		5.06E−24	−7.732451143
*Arid3b*	367092	8:C_005107.4	9.37E−23	−7.636486612
*Dync1li1*	252902	8:NC_005107.4	9.72E−23	−7.635266703
*Daam1*	314212	6:NC_005105.4	5.33E−22	−7.576894143
*CTSE*	LOC001510	1:NC_000001.11	1.27E-20	−7.462898784
*Igsf3*	295325	2: NC_005101.4	5.09E−20	−7.410558702
*Rcn1*	362182	3: NC_005102.4	6.97E−19	−7.307683071
*Fbln5*	29158	6:NC_005105.4	1.75E−18	−7.270057221
*Csrnp1*	363165	8:NC_005107.4	3.35E−16	−7.038893991

**Table 5:** The detailed information of the top 20 upregulated DElncRNA

Upregulated gene name	Gene ID	Location	***Q*** value	Log_**2**_ fold change (Pac/control)
*LOC108353231*	108353231	20:NC_005119.4	3.80E−79	9.808117242
*LOC024465*			2.77E−60	8.102969671
*LOC103693147*	103693147	8:NC_005107.4	1.37E−21	7.462230128
*LOC100910237*	100910237	1:NC_005100.4	5.01E−14	6.709403702
*LOC108352467*	108352467	12:NC_005111.4	4.91E−07	5.513006489
*LOC102555451*	102555451	19:NC_005118.4	6.90E−15	5.431212398
*LOC102547829*	102547829	17:NC_005116.4	4.85E−05	4.928043989
*LXLOC_018745*			8.77E−05	4.834934584
*LXLOC_028716*			9.26E − 05	4.826252341
*LXLOC_022035*			0.000144899	4.751793349
*LOC102553678*	102553678	4:NC_005103.4	0.000159494	4.735398911
*LXLOC_017539*			0.000497496	4.527361782
*LXLOC_024464*			4.36E−12	4.513006489
*LXLOC_001959*			9.75E−07	4.513006489
*LXLOC_009597*			0.000692202	4.461607337
*LXLOC_034027*			0.000988798	4.387475607
*LXLOC_000038*			0.001265955	4.334036348
*LXLOC_034063*			0.00183728	4.249972083
*LOC103690244*	103690244	9:NC_005108.4	0.00243296	4.183544722
*LXLOC_010634*			0.003435335	4.09796899

**Table 6:** The detailed information of the top 20 downregulated DElncRNAs

Downregulated gene name	Gene ID	Location	***Q*** value	Log_**2**_ fold change (Pac/control)
*LXLOC_012126*			8.13E−87	−10.07195601
*LXLOC_011574*			2.94E−134	−9.618850471
*LXLOC_012123*			1.94E−15	−6.954599061
*LXLOC_011573*			1.94E−15	-6.954599061
*LXLOC_034631*			9.80E−14	−6.750309665
*LXLOC_017190*			1.92E−12	−6.577846941
*LXLOC_029452*			6.92E−12	−6.498220766
*LXLOC_025643*			7.61E−12	−6.492168902
*LXLOC_011438*			3.77E-08	−5.840669668
*LXLOC_014875*			4.69E−06	−5.319883525
*LXLOC_001291*			5.37E−05	−4.978846607
*LXLOC_017452*			5.37E−05	−4.978846607
*LXLOC_018867*			0.000187381	−4.77239573
*LXLOC_007757*			0.000187381	−4.77239573
*LXLOC_035549*			2.35E−07	−4.715812201
*LXLOC_015298*			0.000353121	−4.656918512
*LXLOC_021976*			0.000714109	−4.518212241
*LXLOC_015208*			0.001278157	−4.393884106
*LOC102550154*	102550154	17:NC_005116.4	0.001278157	−4.393884106
*LXLOC_012552*			0.001278157	−4.393884106

### Validation of expression changes of DElncRNAs and DEmRNAs by real-time quantitative PCR (qPCR) assay and validation of protein expressions

**Fig. 5:**
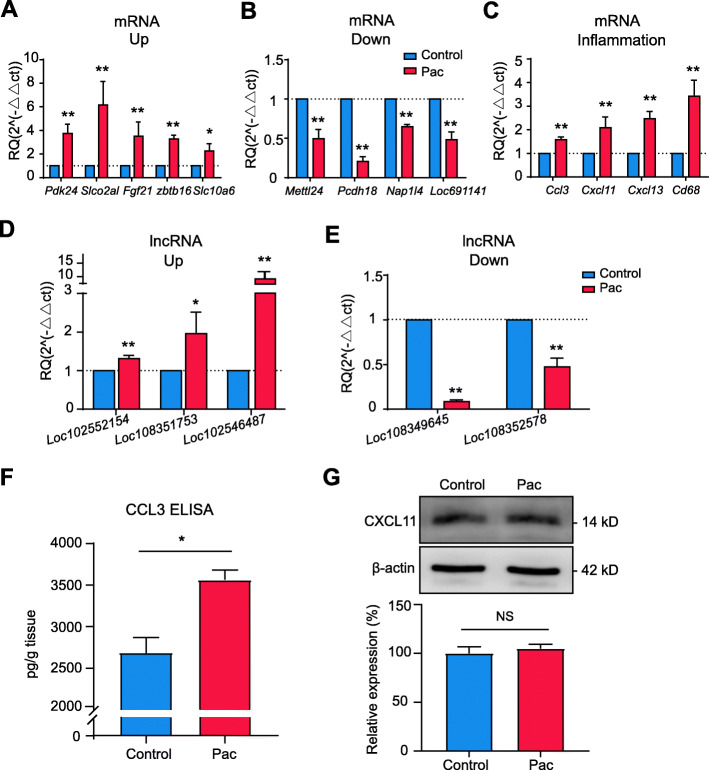
The validation of RNA-Seq dataset by means of qPCR and protein assays. **a**, **b** The expression of 5 randomly selected upregulated DEmRNAs (**a**) and 4 randomly selected downregulated DEmRNAs (**b**) from RNA-Seq dataset validated by qPCR. **c** The expression of some representative genes potentially involved in inflammation and pain response validated by qPCR. **d**, **e** The expression of 3 randomly selected upregulated DElncRNAs (**d**) and 2 randomly selected downregulated DElncRNAs (**e**) from RNA-Seq validated by qPCR. **f** ELISA test of CCL3 protein expression. **g** Western blot test of CXCL11 protein expression. *n* = 6 rats/group. ^*^p < 0.05, ^**^p < 0.01 *vs*. control group. Student’s *t* test was used for statistical analysis

To validate the reliability of the RNA-Seq dataset, 13 DEmRNAs (5 upregulated and 4 downregulated) and 5 DElncRNAs (3 upregulated and 2 downregulated) were randomly selected from the DEGs list for qPCR analysis. qPCR results indicated that the expression of *Pdk24*, *Slco2al*, *Fgf21*, *Zbtb16*, and *Slc10a6* were all significantly upregulated, whereas *Mettl24*, *Pcdh18*, *Nap1l4*, and *L0c69114* were all significantly downregulated in SCDH of Pac group *vs*. control group rats, a result consistent with RNA-Seq dataset (Fig. 5a, b). We further picked up several genes that were well established to be involved in mediating inflammation or pain responses and evaluated their expressions by qPCR. These genes included *Ccl3*, *Cxcl11*, *Cxcl13*, and *CD68*. qPCR results indicated that the expression of these genes was all significantly upregulated, a result consistent with RNA-Seq dataset (Fig. 5c). In addition, qPCR indicated that the expression of 3 DElncRNAs, namely *Loc102552154*, *Loc108351753*, and *Loc102546487* were significantly upregulated and 2 DElncRNAs, namely *Loc108349645* and *Loc108352578* were downregulated, which was also consistent with RNA-Seq dataset (Fig. 5d, e).

We further validated the protein expression of two chemokines, namely, CCL3 and CXCL11, whose gene expression has been confirmed by qPCR. We found that the protein expression (tested by ELISA) of CCL3 is significantly increased in spinal cord tissue from paclitaxel-treated rats compared with control rats (Fig. 5f). This result is consistent with the RNA-Seq and qPCR results. However, we did not observe any significant upregulation of the protein expression of CXCL11 by paclitaxel treatment (Fig. 5g).

### Function and pathway analysis of the identified DElncRNAs and DEmRNAs

**Fig. 6:**
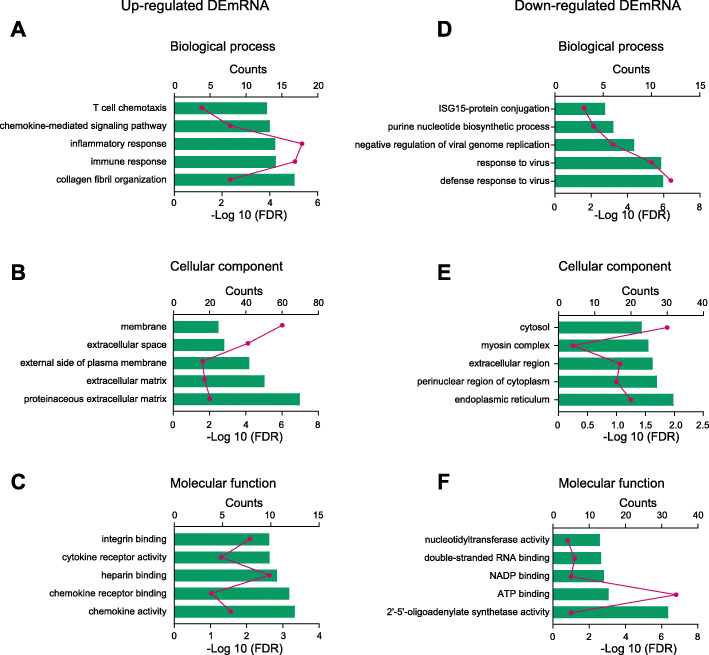
GO pathway analysis for DEmRNAs. **a**–**c** The top 5 significant biological processes, molecular functions, and cellular components of upregulated DEmRNAs. **d**–**f** The top 5 significant biological processes, molecular functions, and cellular components of downregulated DEmRNAs. The dotted line indicated a *p* value of 0.001

To further investigate the molecular mechanisms underlying PIPN, we performed GO analysis of the DEmRNAs that were identified. Results obtained from GO analysis of upregulated DEmRNAs indicated that the most significantly enriched biological process (BP) was response to collagen fibril organization, immune response, inflammatory response, chemokine-mediated signaling pathway and T cell chemotaxis, etc. (Fig. 6a, Additional file 3: Suppl. Table 3). The most significantly enriched cellular component (CC) of upregulated DEmRNAs was proteinaceous extracellular matrix, extracellular matrix, external side of plasma membrane, extracellular space and membrane, etc. (Fig. 6b, Additional file 4: Suppl. Table 4). The most significantly enriched molecular function (MF) of upregulated DEmRNAs was chemokine activity, chemokine receptor binding, cytokine receptor activity, integrin binding, etc. (Fig. 6c, Additional file 5: Suppl. Table 5). In contrast, the most significantly enriched BP of downregulated DEmRNAs was defense response to virus, response to virus, negative regulation of viral genome replication, purine nucleotide biosynthetic process, ISG15-protein conjugation, etc. (Fig. 6d, Additional file 3: Suppl. Table 3). The most significantly enriched CC of downregulated DEmRNAs was endoplasmic reticulum, perinuclear region of cytoplasm, extracellular region, myosin complex, cytosol, etc. (Fig. 6e, Additional file 4: Suppl. Table 4). The most significantly enriched MF of downregulated DEmRNAs was 2′–5′-oligoadenylate synthetase activity, ATP binding, NADP binding, double-stranded RNA binding, nucleotidyltransferase activity, etc. (Fig. 6f, Additional file 5: Suppl. Table 5).

We also performed Gene Set Enrichment Analysis (GSEA) to further evaluate the enriched pathways from GO analysis. The top 12 most significantly upregulated pathways by paclitaxel treatment deduced from GSEA analysis (NES > 1.0, FDR ≤ 0.25) are shown in Suppl. Fig. 1. The GSEA analysis indicates that immune responses predominate among these pathways.

**Fig. 7:**
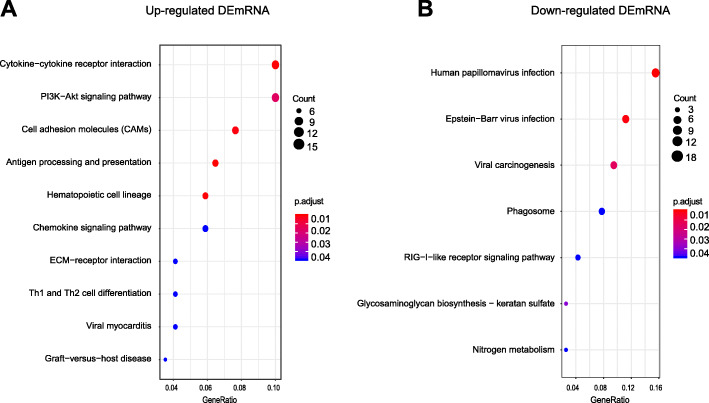
KEGG pathway analysis of DEmRNAs. **a** Bubble plots showing the top 10 significant pathways for upregulated DEmRNAs. **b** Bubble plots showing the top 7 significant pathways for downregulated DEmRNAs. Larger bubbles indicate higher number of genes. The color of each bubble reflects the significance (*p* value)

Next, we carried out KEGG pathway analysis of the DEmRNAs. As shown in Fig. 7a (Additional file 6: Suppl. Table 6), the upregulated DEmRNAs were mainly involved in cytokine-cytokine receptor interaction, PI3K-Akt signaling pathway, cell adhesion molecules (CAMs), antigen processing and presentation, hematopoietic cell lineage, etc. The downregulated DEmRNAs mainly involved human papillomavirus infection, Epstein-barr virus infection, viral carcinogenesis, etc.

**Fig. 8:**
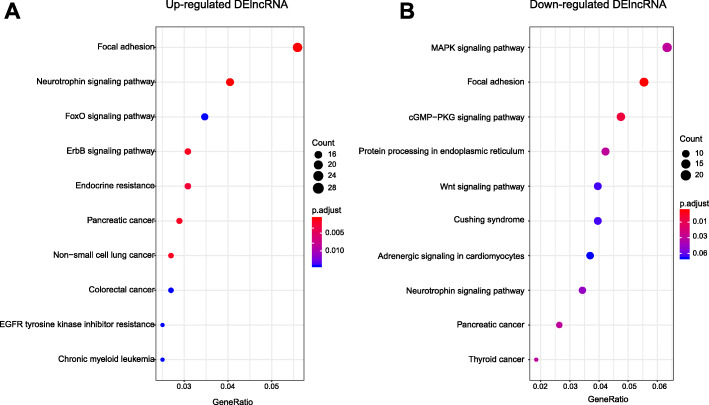
KEGG pathway analysis of DElncRNAs. **a** Bubble plots showing the top 10 significant pathways for upregulated DElncRNAs. **b** Bubble plots showing the top 10 significant pathways for downregulated DElncRNAs. Larger bubbles indicate higher number of genes. The color of each bubble reflects the significance (*p* value)

KEGG analysis of the upregulated DElncRNAs in SCDH of PIPN model rat were primarily enriched in focal adhesion, neurotrophin signaling pathway, foxo signaling pathway, etc. (Fig. 8a, Additional file 7: Suppl. Table 7). The downregulated DElncRNAs were primarily involved in MAPK signaling pathway, focal adhesion, cGMP-PKG signaling pathway, etc. (Fig. 8b, Additional file 7: Suppl. Table 7).

**Fig. 9:**
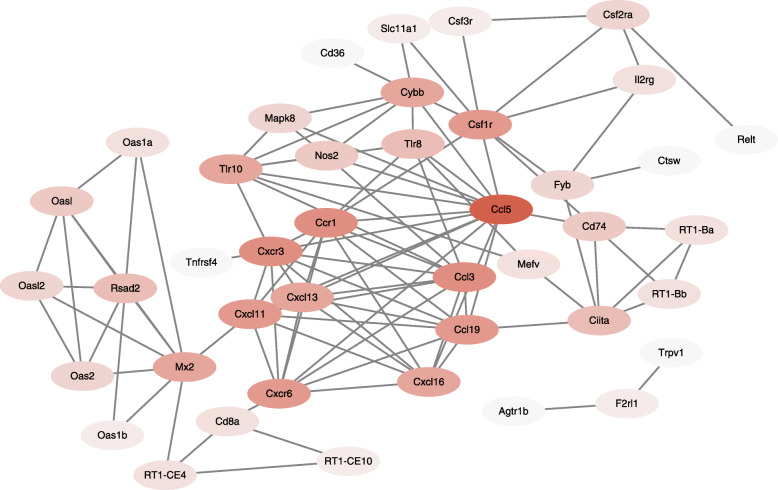
PPI network analysis of genes involved in immune response-related pathways in SCDH of PIPN model rats. Genes related with immune response-related pathways identified by GO and KEGG analysis were subject to PPI network analysis. Deeper colors reflect more interactions and vice versa

We were especially interested in pathways involved in immunological responses, since these pathways may get involved in mediating neuroinflammation and pain mechanisms in SCDH of PIPN model rats. Therefore, we continued to perform PPI analysis of the genes that are related to immune-related biological process we identified from GO and KEGG analysis, including T cell chemotaxis, chemokine-mediated signaling pathway, inflammatory response, immune response, and chemokine receptor bind. In total, 42 genes were identified thereafter and the major hub genes deduced from PPI analysis consisted of *Ccl5*, *Ccl3*, *Ccl19*, *Cxcr3*, *Cxcl13*, *Cxcl11*, *Cxcr6*, *Tlr10*, *Mx2*, *Cybb*, *Csf1r*, etc. (Fig. 9).

### Comparison of RNA-Seq dataset of PIPN rat model with other published datasets of neuropathic pain models

**Fig. 10:**
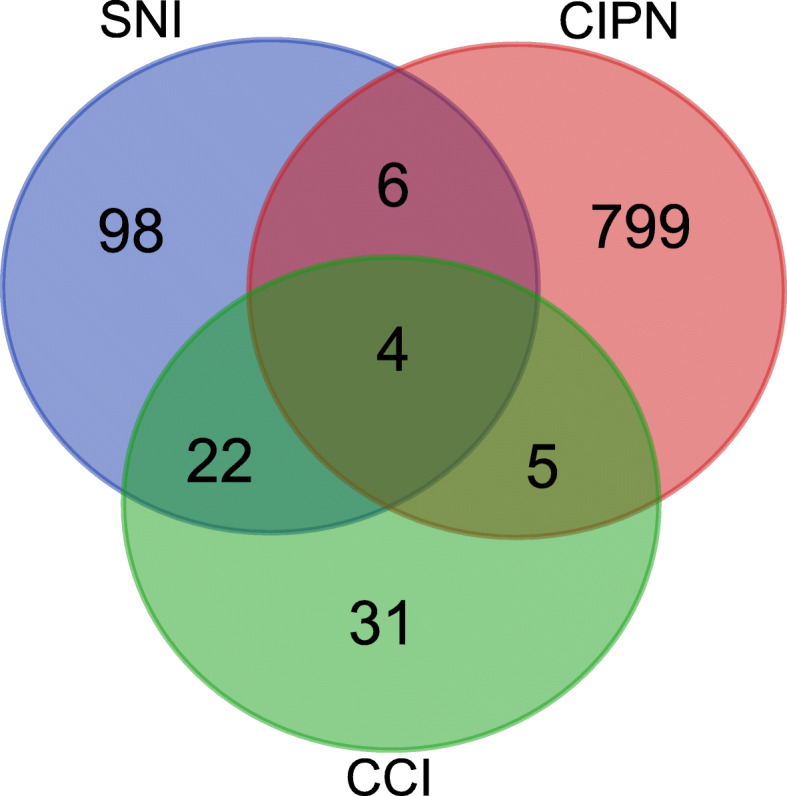
Comparison of the PIPN RNA-Seq dataset with two other microarray/RNA-Seq datasets of neuropathic pain models. Venn diagram showing the overlapping of DEGs in DRGs from PIPN rat model with rat model of SNI and CCI neuropathic pain

We then compared the PIPN RNA-Seq dataset with microarray/RNA-Seq datasets from rat CCI and SNI models, two well-known neuropathic pain models. The same criteria (fold change ≥2, *q* ≤ 0.01) were imposed upon both SNI and CCI microarray datasets to screen DEmRNAs. The DEmRNAs of PIPN model had 9 and 10 genes overlapping with CCI and SNI models, respectively (Fig. 10 and Additional file 8: Suppl. Table 8). In addition, all three groups had a core set of 4 overlapping genes (Fig. 10 and Additional file 9: Suppl. Table 9).

### Competing endogenous RNA (ceRNA) analysis of DElncRNAs, miRNAs, and DEmRNAs

**Fig. 11:**
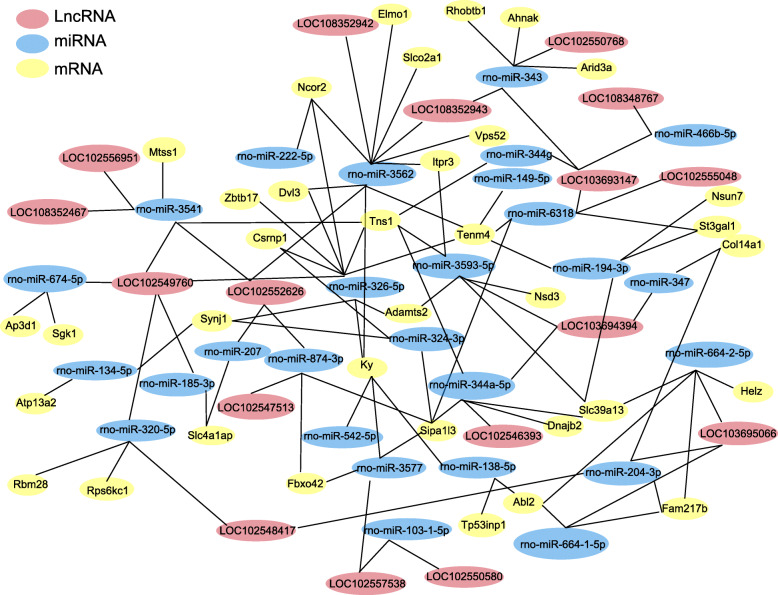
CeRNA network analysis of SCDH from the rat model of PIPN. The red, blue, and yellow ellipse nodes indicate DElncRNA, miRNA, and DEmRNA, respectively

The translation of mRNAs may be affected by lncRNAs via sponging miRNAs. We then constructed a competitive endogenous network of lncRNA-miRNA-mRNA to predict the mechanisms of lncRNAs and the potential miRNA targets that they may affect. The competitive endogenous RNA (ceRNA) network was created based upon expression consistency, sequence similarity, and maximum binding free energy. The network analysis yielded a result of 17 DElncRNAs, 27 miRNAs, and 35 DEmRNAs with 110 edges (Fig. 11). The network analysis further identified that the major miRNAs that were competitively bound by ceRNAs included miR-3562, miR-3593-5p, miR-326-5p, miR-344a-5p, miR-3541, etc. (Fig. 11).

## Discussion

In the present study, we successfully established the rat PIPN model. We examined the gene expression profiles with the focus on mRNA and lncRNA in lumber SCDH of the rat PIPN model and control rats through RNA-Seq. We found a number of differentially expressed mRNAs and lncRNAs. We further validated their expressions via qPCR and protein assay and looked into the molecular functions, cellular component, and the most significantly enriched biological processes of these DEGs by applying bioinformatic analysis. We found that a sizeable proportion of these DEGs were exclusively involved in inflammatory processes and immune responses. In addition, ceRNA analysis further identified direct or indirect regulatory relationship may exist between these DEmRNAs and DElncRNAs under conditions of PIPN.

It is well established that astrocytes and microglia from SCDH play crucial roles in promoting neuroinflammation and central sensitization during chronic pain [46–48]. Up to date, mounting evidence suggested that both astrocytes and microglia are important contributors to pain mechanisms of PIPN. These glia cells make their contributions by promoting neuron-glia communications, increasing neuronal excitability, releasing pro-inflammatory cytokines/chemokines, etc. in PIPN model animals [12–14]. In the present study, we found that PIPN model rats showed significant activation of both microglia and astrocytes in SCDH. However, an earlier study by Zheng et al. report no increment of spinal microglia activation associated with paclitaxel neuropathy using the same rat model as we used. In contrast, some recent studies reported obvious spinal microglia activation using the same paclitaxel model as we used in the present study [49]. Moreover, our recent study, which used the same paclitaxel model, also identified significant spinal microglia activation after paclitaxel treatment and we further evaluated the effect of electroacupuncture on alleviating microglia activation in spinal cord [11]. These findings are all consistent with our present study. Recently, mounting evidence indicates that spinal microglia is clearly activated after paclitaxel treatment, although different dosages of paclitaxel or treatment regime may vary in these studies [44, 50–52]. More importantly, inhibition of microglia activity further alleviated paclitaxel-induced neuropathic pain [53], indicating a critical role of spinal microglia in mediating PIPN. At this moment, we have no clues why the study by Zheng et al. found no microglial activation in the spinal cord. But it has become more and more apparent nowadays that spinal microglia are activated after paclitaxel treatment and played an important role in mediating neuroinflammation and pain response. The sustained glial cell activation in SCDH can produce an array of pro-inflammatory mediators, including cytokines or chemokines, in spinal cord [54]. These pro-inflammatory mediators make important contributions to the pain mechanisms of PIPN by causing neuroinflammation and promoting central sensitization [13, 14]. Therefore, we performed RNA-Seq of SCDH of PIPN model rats, with the purpose to explore the pain mechanisms underlying PIPN.

KEGG analysis unraveled several signaling pathways enriched in SCDH of PIPN model rats. Among these pathways, cytokine-cytokine receptor, chemokine signaling, and PI3K-AKT pathways especially attracted our attention since they have been implicated in mediating chronic pain. Cytokine-cytokine receptor and chemokine signaling pathways are important for mediating neuron-glia crosstalk, which plays a critical role in glial and neuron activation, cytokine production, neuroinflammation, and pathogenesis of chronic pain. One of the major mechanisms for conducting the crosstalk is via cytokine or chemokine release from glia and the binding to corresponding receptors expressed in neurons or vice versa [48, 55]. Therefore, cytokine-cytokine receptor pathway and chemokine signaling pathway are activated among neurons, astrocytes, and microglia to mediate the neuro-glia crosstalk during chronic pain. Paclitaxel can induce the production of certain pro-inflammatory cytokines in spinal cord, including IL-17, TNF-α, IL-1β, etc. [12–14]. These cytokines can bind with their receptors to enhance glutamatergic activity and suppress inhibitory synaptic transmission, which results in neuronal hyperexcitability in SCDH and contributes to chronic pain [13, 14]. Spinal PI3K-AKT pathway has also been demonstrated to mediate chronic pain [56]. PI3Kγ and pAKT have been shown to be predominantly expressed in spinal neurons and astrocytes and a minority of microglia [57], where the pathway activation may take place. Therefore, these results suggest that targeting cytokine-cytokine receptor interaction, chemokine signaling, or PI3K-Akt signaling may be novel strategies to treat PIPN.

We noticed that the most significantly enriched biological process of downregulated DEmRNAs was defense response to virus, response to virus, negative regulation of viral genome replication, etc. At this stage, we have no idea how these processes might be related with paclitaxel treatment. But evidence indicates that, in addition to its anti-tubulin property, paclitaxel also exerts remarkable effects on innate immunity [58–60], which acts as the first line to defend against viral invasion or replication before adaptive immune system kicks in. Therefore, we postulated that paclitaxel might act on the immune system in the spinal cord to modulate the expression of genes related with response to virus. But how these biological processes might be related with paclitaxel-induced neuropathic pain is unknown and still needs further investigation.

We found that *Ccl3* gene expression is increased in spinal cord dorsal horn of paclitaxel-treated rats by RNA-Seq and qPCR. ELISA test further validated the upregulation of CCL3 protein expression. A previous study by Ochi-ishi et al. indicated that intrathecal injection of an antibody against CCL3 significantly attenuated paclitaxel-induced mechanical allodynia in rats [61]. However, only gene expression but not protein expression of CCL3 was tested in that study. Therefore, our study further proved the upregulation of CCL3 protein expression in spinal cord dorsal horn. Our study, together with the study by Ochi-ishi et al., suggested targeting CCL3 may offer novel therapeutic approach for paclitaxel-induced neuropathic pain.

*Cxcl13* gene is among one of the most upregulated pain-related genes in SCDH. CXCL13 is a B lymphocyte chemoattractant with CXCR5 as its receptor. A recent study found that in a mouse SNL neuropathic pain model, CXCL13 was persistently upregulated in spinal cord neurons, resulting in spinal astrocyte activation via CXCR5. *Cxcl13* knockdown via shRNA or genetic knockout of *Cxcr5* significantly attenuates spinal cord astrocyte activation and relieves SNL-induced neuropathic pain [62]. Recent work further identified that CXCL13/CXCR5 contributes to diabetes-induced mechanical hypersensitivities by activating pERK, pSTAT3, and pAKT pathways and promoting TNF-α and IL-6 production in SCDH [63]. Our recent work identified that *Cxcl13* gene is significantly upregulated in SCDH of a rat model of complex regional pain syndrome type-I [26, 64]. These studies all support an important role of CXCL13/CXCR5 signaling in mediating chronic pain. Therefore, our findings suggest that CXCL13 may be a novel therapeutic target for PIPN.

LncRNA plays a vital role in regulating the expression of coding genes [65, 66]. Several LncRNAs have been identified to regulate certain pain-related gene expression in chronic pain conditions, thus contributing to regulatory mechanisms of chronic pain [20, 67, 68]. A large number of DElncRNAs have been identified in DRGs or spinal cord of several neuropathic pain models [69–72]. However, studies about lncRNA identification and function under PIPN condition are still lacking. Therefore, we attempted to screen potential lncRNAs and identify their potential biological functions in PIPN model rats. KEGG analysis of DElncRNAs suggested that they are primarily involved in neurotrophin signaling pathway. The neurotrophin signaling, when activated by neurotrophins through binding to Trk receptors, results in the recruitment of a series of signaling proteins [73]. These signaling proteins activate downstream intracellular signaling pathways, including ERK1/2 and NF-κB pathway. ERK1/2 signaling was recently found to be upregulated and contributed to PIPN by enhancing Nav1.7 expression in DRGs of a rat model of PIPN [74]. Besides, NF-κB signaling was shown to be upregulated in the spinal cord of a rat PIPN model [75]. These results suggest that lncRNAs may modulate mRNA expression involved in neurotrophin signaling pathway during PIPN, which may consequently affect downstream ERK1/2 s and NF-κB signaling. Thus, unravelling the mechanisms underlying how lncRNAs participate in PIPN may help to develop novel therapeutic approaches.

In the present study, we used saline as a control. However, the paclitaxel formulation we used for model establishment contains ingredients of Cremophor EL and ethanol as vehicle. It is reported that Cremophor EL may be toxic and could cause neurotoxicity per se [76]. In order to exclude the unwanted side effects of Cremophor EL and ethanol on pain mechanisms in our study, we further performed experiments to study the effects of Cremophor EL and ethanol on pain thresholds. We found that application of equal amount of Cremophor EL and ethanol as in paclitaxel formulation application produced no effect on animal mechanical pain thresholds (Suppl. Fig. 2A-C). Besides, Cremophor EL and ethanol produced no effect on astrocyte and microglia activation in spinal cord dorsal horn (Suppl. Fig. 2D-G). These results suggest Cremophor EL and ethanol did not elicit pain response nor produce any effect on neuroinflammation in SCDH. We have to acknowledge that these experiments cannot completely rule out potential side effects of Cremophor EL and ethanol on other targets or signaling pathways. However, it should be noted that using saline alone as a control was not uncommon among literatures [49, 77–79]. It is known that paclitaxel formulation with Cremophor EL and ethanol as vehicle (like the one we obtained from Hospira Australia Pty. Ltd., Australia) is still the predominant pharmaceutical products used for chemotherapy among patients. Patients are infused with the whole formulation diluted in saline for chemotherapy. Cremophor EL per se may cause certain neurotoxic effects, including axonal degeneration and demyelination [76]. The combination of Cremophor EL with paclitaxel may further promote paclitaxel’s neurotoxicity [76]. So the observed CIPN among patients may be due to the overall toxic effects of paclitaxel combined with Cremophor EL. Therefore, using saline alone as a control have its own merit such that it could help to understand the overall effects of the whole paclitaxel formulation, which the patients were usually treated with, on the sensory nerve system.

## Conclusions

The RNA-Seq of the present study provides a landscape of expression changes of mRNAs and lncRNAs in SCDH of a rat model of PIPN. Pathway and functional analysis further identified a number of DEGs and signaling pathways in SCDH that may possibly contribute to the neuroinflammation and pain response of PIPN. Our study may provide some novel insights into understanding the molecular mechanisms underlying PIPN, which may help to develop new treatments for PIPN.

## Supplementary Information

**Additional file 1.**

**Additional file 2.**

**Additional file 3.**

**Additional file 4.**

**Additional file 5.**

**Additional file 6.**

**Additional file 7.**

**Additional file 8.**

**Additional file 9.**

**Additional file 10: Suppl. Fig. 1.** Gene Set Enrichment Analysis (GSEA) of enriched pathways from GO analysis. GSEA analysis of RNA-Seq dataset of PIPN model rats using well-defined gene sets derived from GO analysis. The top 12 most significantly upregulated pathways by paclitaxel treatment deduced from GSEA analysis was listed, with NES > 1.0 and FDR ≦ 0.25 as cut off threshold.

**Additional file 11: Suppl. Fig. 2.** The comparison of the effects of 0.9% saline and Cremophor EL/ethanol on mechanical pain threshold and spinal glial activation in rats. (A) Experimental protocol for saline or Cremophor EL/ethanol treatment. Saline or Cremophor EL/ethanol (1:5 diluted in saline, according to dilution ratio of paclitaxel formulation used for PIPN model establishment) was injected (i.p.) with a volume of 0.5 ml/250 g body weight into rats at time points as indicated. (B) Effects of saline or Cremophor EL/ethanol on mechanical pain threshold. (C) Normalized AUC analysis of panel (A). (D) Representative immunofluorescence images indicating OX42 antibody staining of spinal cord from saline- and Cremophor EL/ethanol-treated groups. (E) Percentage of OX42 positively staining area in each observation field (left panel). Summary of the normalized % increase in fluorescence intensity of OX42 immunostaining in each observation field (right panel). The value of each group was normalized to that of saline group. (F) Representative immunofluorescence images indicating GFAP antibody staining of spinal cord from saline- and Cremophor EL/ethanol-treated groups. (G) Percentage of GFAP positively stained area in each observation field (left panel). Summary of the normalized % increase in fluorescence intensity of GFAP immunostaining in each observation field (right panel). The value of each group was normalized to that of saline group. n = 5 rats/group. NS: no significance. Student’s *t* test was used for statistical analysis.

## Data Availability

The key data are contained in the figures, tables, and additional files. The datasets used and/or analyzed during this study can be further obtained from the corresponding author on reasonable request.

## Abbreviations

PIPN: Paclitaxel-induced peripheral neuropathy
lncRNA: Long noncoding RNA
RNA-Seq: RNA sequencing
SCDH: Spinal cord dorsal horn
DEmRNA: Differentially expressed mRNA
DElncRNA: Differentially expressed lncRNA
CIPN: Chemotherapy-induced peripheral neuropathy
Pac: Paclitaxel
qPCR: Real-time quantitative PCR
DRG: Dorsal root ganglion
i.p.: Intraperitoneally
PWT: Paw withdrawal threshold
PWL: Paw withdrawal latency
QC: Quality control
KEGG: Kyoto Encyclopedia of Genes and Genomes
GO: Gene ontology
BP: Biological processes
CC: Cellular component
MF: Molecular function
PPI: Protein-protein interaction
SNI: Spared nerve injury
CCI: Chronic constriction injury
GEO: Gene Expression Omnibus
STRING: Search Tool for the Retrieval of Interacting Genes
ceRNA: Competing endogenous RNA
DEG: Differentially expressed gene

## References

[1] Banach M, Juranek JK, Zygulska AL (2017). Chemotherapy-induced neuropathies-a growing problem for patients and health care providers. Brain Behav.

[2] Mody MD, Gill HS, Saba NF (2016). The evolving and future role of taxanes in squamous cell carcinomas of the head and neck: a review. JAMA Otolaryngol Head Neck Surg.

[3] Fojo T, Menefee M (2007). Mechanisms of multidrug resistance: the potential role of microtubule-stabilizing agents. Ann Oncol.

[4] Starobova H, Vetter I (2017). Pathophysiology of chemotherapy-induced peripheral neuropathy. Front Mol Neurosci.

[5] Brewer JR, Morrison G, Dolan ME, Fleming GF (2016). Chemotherapy-induced peripheral neuropathy: current status and progress. Gynecol Oncol.

[6] Postma TJ, Vermorken JB, Liefting AJ, Pinedo HM, Heimans JJ (1995). Paclitaxel-induced neuropathy. Ann Oncol.

[7] Meng J, Zhang Q, Yang C, Xiao L, Xue Z, Zhu J (2019). Duloxetine, a balanced serotonin-norepinephrine reuptake inhibitor, improves painful chemotherapy-induced peripheral neuropathy by inhibiting activation of p38 MAPK and NF-κB. Front Pharmacol.

[8] da Costa R, Passos GF, Quintao NLM, Fernandes ES, Maia J, Campos MM (2020). Taxane-induced neurotoxicity: pathophysiology and therapeutic perspectives. Br J Pharmacol.

[9] Flatters SJL, Dougherty PM, Colvin LA (2017). Clinical and preclinical perspectives on chemotherapy-induced peripheral neuropathy (CIPN): a narrative review. Br J Anaesth.

[10] Sisignano M, Baron R, Scholich K, Geisslinger G (2014). Mechanism-based treatment for chemotherapy-induced peripheral neuropathic pain. Nat Rev Neurol.

[11] Li Y, Yin C, Li X, Liu B, Wang J, Zheng X (2019). Electroacupuncture alleviates paclitaxel-induced peripheral neuropathic pain in rats via suppressing TLR4 signaling and TRPV1 upregulation in sensory neurons. Int J Mol Sci.

[12] Yan X, Li F, Maixner DW, Yadav R, Gao M, Ali MW (2019). Interleukin-1beta released by microglia initiates the enhanced glutamatergic activity in the spinal dorsal horn during paclitaxel-associated acute pain syndrome. Glia.

[13] Liu X, Tonello R, Ling Y, Gao YJ, Berta T (2019). Paclitaxel-activated astrocytes produce mechanical allodynia in mice by releasing tumor necrosis factor-α and stromal-derived cell factor 1. J Neuroinflammation.

[14] Luo H, Liu HZ, Zhang WW, Matsuda M, Lv N, Chen G (2019). Interleukin-17 regulates neuron-glial communications, synaptic transmission, and neuropathic pain after chemotherapy. Cell Rep.

[15] Yang C, Wu K, Wang S, Wei G (2018). Long non-coding RNA XIST promotes osteosarcoma progression by targeting YAP via miR-195-5p. J Cell Biochem.

[16] She K, Yan H, Huang J, Zhou H, He J (2018). miR-193b availability is antagonized by LncRNA-SNHG7 for FAIM2-induced tumour progression in non-small cell lung cancer. Cell Prolif.

[17] Wu S, Bono J, Tao YX (2019). Long noncoding RNA (lncRNA): a target in neuropathic pain. Expert Opin Ther Targets.

[18] Briggs JA, Wolvetang EJ, Mattick JS, Rinn JL, Barry G (2015). Mechanisms of long non-coding RNAs in mammalian nervous system development, plasticity, disease, and evolution. Neuron.

[19] Baskozos G, Dawes JM, Austin JS, Antunes-Martins A, McDermott L, Clark AJ (2019). Comprehensive analysis of long noncoding RNA expression in dorsal root ganglion reveals cell-type specificity and dysregulation after nerve injury. Pain.

[20] Zhao X, Tang Z, Zhang H, Atianjoh FE, Zhao JY, Liang L (2013). A long noncoding RNA contributes to neuropathic pain by silencing Kcna2 in primary afferent neurons. Nat Neurosci.

[21] Wang S, Xu H, Zou L, Xie J, Wu H, Wu B (2016). LncRNA uc.48+ is involved in diabetic neuropathic pain mediated by the P2X3 receptor in the dorsal root ganglia. Purinergic Signal.

[22] Yu W, Zhao GQ, Cao RJ, Zhu ZH, Li K (2017). LncRNA NONRATT021972 was associated with neuropathic pain scoring in patients with type 2 diabetes. Behav Neurol.

[23] Li Z, Li X, Chen X, Li S, Ho IHT, Liu X (2019). Emerging roles of long non-coding RNAs in neuropathic pain. Cell Prolif.

[24] Zhang H, Li Y, de Carvalho-Barbosa M, Kavelaars A, Heijnen CJ, Albrecht PJ (2016). Dorsal root ganglion infiltration by macrophages contributes to paclitaxel chemotherapy-induced peripheral neuropathy. J Pain.

[25] Polomano RC, Mannes AJ, Clark US, Bennett GJ (2001). A painful peripheral neuropathy in the rat produced by the chemotherapeutic drug, paclitaxel. Pain.

[26] Yin C, Hu Q, Liu B, Tai Y, Zheng X, Li Y (2019). Transcriptome profiling of dorsal root ganglia in a rat model of complex regional pain syndrome type-I reveals potential mechanisms involved in pain. J Pain Res.

[27] Chai W, Tai Y, Shao X, Liang Y, Zheng GQ, Wang P (2018). Electroacupuncture alleviates pain responses and inflammation in a rat model of acute gout arthritis. Evid Based Complement Alternat Med.

[28] Dixon WJ (1980). Efficient analysis of experimental observations. Annu Rev Pharmacol Toxicol.

[29] Chaplan SR, Bach FW, Pogrel JW, Chung JM, Yaksh TL (1994). Quantitative assessment of tactile allodynia in the rat paw. J Neurosci Methods.

[30] Hu Q, Wang Q, Wang C, Tai Y, Liu B, Shao X (2019). TRPV1 channel contributes to the behavioral hypersensitivity in a rat model of complex regional pain syndrome type 1. Front Pharmacol.

[31] Li R, Li Y, Kristiansen K, Wang J (2008). SOAP: short oligonucleotide alignment program. Bioinformatics (Oxford, England).

[32] Kim D, Langmead B, Salzberg SL (2015). HISAT: a fast spliced aligner with low memory requirements. Nat Methods.

[33] Benelli M, Pescucci C, Marseglia G, Severgnini M, Torricelli F, Magi A (2012). Discovering chimeric transcripts in paired-end RNA-seq data by using EricScript. Bioinformatics (Oxford, England).

[34] Shen S, Park JW, Lu ZX, Lin L, Henry MD, Wu YN (2014). rMATS: robust and flexible detection of differential alternative splicing from replicate RNA-Seq data. Proc Natl Acad Sci U S A.

[35] Langmead B, Salzberg SL (2012). Fast gapped-read alignment with Bowtie 2. Nat Methods.

[36] Li B, Dewey CN (2011). RSEM: accurate transcript quantification from RNA-Seq data with or without a reference genome. BMC Bioinformatics.

[37] Love MI, Huber W, Anders S (2014). Moderated estimation of fold change and dispersion for RNA-seq data with DESeq2. Genome Biol.

[38] Chen W, Ding H, Zhou X, Lin H, Chou KC (2018). iRNA(m6A)-PseDNC: identifying N(6)-methyladenosine sites using pseudo dinucleotide composition. Anal Biochem.

[39] Liu B, Tai Y, Liu B, Caceres AI, Yin C, Jordt SE (2019). Transcriptome profiling reveals Th2 bias and identifies endogenous itch mediators in poison ivy contact dermatitis. JCI Insight.

[40] Livak KJ, Schmittgen TD (2001). Analysis of relative gene expression data using real-time quantitative PCR and the 2(-Delta Delta C(T)) Method. Methods (San Diego, Calif).

[41] Yin C, Liu B, Li Y, Li X, Wang J, Chen R (2020). IL-33/ST2 induces neutrophil-dependent reactive oxygen species production and mediates gout pain. Theranostics.

[42] Franceschini A, Szklarczyk D, Frankild S, Kuhn M, Simonovic M, Roth A (2013). STRING v9.1: protein-protein interaction networks, with increased coverage and integration. Nucleic Acids Res.

[43] Shannon P, Markiel A, Ozier O, Baliga NS, Wang JT, Ramage D (2003). Cytoscape: a software environment for integrated models of biomolecular interaction networks. Genome Res.

[44] Wu J, Hocevar M, Bie B, Foss JF, Naguib M (2019). Cannabinoid type 2 receptor system modulates paclitaxel-induced microglial dysregulation and central sensitization in rats. J Pain.

[45] Peters CM, Jimenez-Andrade JM, Jonas BM, Sevcik MA, Koewler NJ, Ghilardi JR (2007). Intravenous paclitaxel administration in the rat induces a peripheral sensory neuropathy characterized by macrophage infiltration and injury to sensory neurons and their supporting cells. Exp Neurol.

[46] Milligan ED, Watkins LR (2009). Pathological and protective roles of glia in chronic pain. Nat Rev Neurosci.

[47] Hu Q, Zheng X, Li X, Liu B, Yin C, Li Y (2020). Electroacupuncture alleviates mechanical allodynia in a rat model of complex regional pain syndrome type-I via suppressing spinal CXCL12/CXCR4 signaling. J Pain.

[48] Donnelly CR, Andriessen AS, Chen G, Wang K, Jiang C, Maixner W (2020). Central nervous system targets: glial cell mechanisms in chronic pain. Neurotherapeutics.

[49] Zhao YX, Yao MJ, Liu Q, Xin JJ, Gao JH, Yu XC (2020). Electroacupuncture treatment attenuates paclitaxel-induced neuropathic pain in rats via inhibiting spinal glia and the TLR4/NF-kappaB pathway. J Pain Res.

[50] Wang J, Jiang C, Ba X, Yang S, Wu J, Huang Z, et al. Selective activation of metabotropic glutamate receptor 7 blocks paclitaxel-induced acute neuropathic pain and suppresses spinal glial reactivity in rats. Psychopharmacology (Berl). 2021;238(1):107–19. 10.1007/s00213-020-05662-1 33089875

[51] Beh ST, Kuo YM, Chang WW, Wilder-Smith E, Tsao CH, Tsai CH (2019). Preventive hypothermia as a neuroprotective strategy for paclitaxel-induced peripheral neuropathy. Pain.

[52] Liu X, Tonello R, Ling Y, Gao YJ, Berta T (2019). Paclitaxel-activated astrocytes produce mechanical allodynia in mice by releasing tumor necrosis factor-alpha and stromal-derived cell factor 1. J Neuroinflammation.

[53] Saika F, Matsuzaki S, Kobayashi D, Ideguchi Y, Nakamura TY, Kishioka S (2020). Chemogenetic regulation of CX3CR1-expressing microglia using Gi-DREADD exerts sex-dependent anti-allodynic effects in mouse models of neuropathic pain. Front Pharmacol.

[54] Ji RR, Chamessian A, Zhang YQ (2016). Pain regulation by non-neuronal cells and inflammation. Science (New York, NY).

[55] Jiang BC, Liu T, Gao YJ (2020). Chemokines in chronic pain: cellular and molecular mechanisms and therapeutic potential. Pharmacol Ther.

[56] Chen SP, Zhou YQ, Liu DQ, Zhang W, Manyande A, Guan XH (2017). PI3K/Akt pathway: a potential therapeutic target for chronic pain. Curr Pharm Des.

[57] Guan X, Fu Q, Xiong B, Song Z, Shu B, Bu H (2015). Activation of PI3Kgamma/Akt pathway mediates bone cancer pain in rats. J Neurochem.

[58] Javeed A, Ashraf M, Riaz A, Ghafoor A, Afzal S, Mukhtar MM (2009). Paclitaxel and immune system. Eur J Pharm Sci.

[59] Zeng QZ, Yang F, Li CG, Xu LH, He XH, Mai FY (2019). Paclitaxel enhances the innate immunity by promoting NLRP3 inflammasome activation in macrophages. Front Immunol.

[60] Wanderley CW, Colón DF, Luiz JPM, Oliveira FF, Viacava PR, Leite CA (2018). Paclitaxel reduces tumor growth by reprogramming tumor-associated macrophages to an M1 profile in a TLR4-dependent manner. Cancer Res.

[61] Ochi-ishi R, Nagata K, Inoue T, Tozaki-Saitoh H, Tsuda M, Inoue K (2014). Involvement of the chemokine CCL3 and the purinoceptor P2X7 in the spinal cord in paclitaxel-induced mechanical allodynia. Mol Pain.

[62] Jiang BC, Cao DL, Zhang X, Zhang ZJ, He LN, Li CH (2016). CXCL13 drives spinal astrocyte activation and neuropathic pain via CXCR5. J Clin Invest.

[63] Liu S, Liu X, Xiong H, Wang W, Liu Y, Yin L (2019). CXCL13/CXCR5 signaling contributes to diabetes-induced tactile allodynia via activating pERK, pSTAT3, pAKT pathways and pro-inflammatory cytokines production in the spinal cord of male mice. Brain Behav Immun.

[64] Chen R, Yin C, Hu Q, Liu B, Tai Y, Zheng X (2020). Expression profiling of spinal cord dorsal horn in a rat model of complex regional pain syndrome type-I uncovers potential mechanisms mediating pain and neuroinflammation responses. J Neuroinflammation.

[65] Kopp F, Mendell JT (2018). Functional classification and experimental dissection of long noncoding RNAs. Cell.

[66] Quinn JJ, Chang HY (2016). Unique features of long non-coding RNA biogenesis and function. Nat Rev Genet.

[67] Li G, Jiang H, Zheng C, Zhu G, Xu Y, Sheng X (2017). Long noncoding RNA MRAK009713 is a novel regulator of neuropathic pain in rats. Pain.

[68] Wen J, Yang Y, Wu S, Wei G, Jia S, Hannaford S (2020). Long noncoding RNA H19 in the injured dorsal root ganglion contributes to peripheral nerve injury-induced pain hypersensitivity. Transl Perioper Pain Med.

[69] Jiang BC, Sun WX, He LN, Cao DL, Zhang ZJ, Gao YJ (2015). Identification of lncRNA expression profile in the spinal cord of mice following spinal nerve ligation-induced neuropathic pain. Mol Pain.

[70] Duran RC, Yan H, Zheng Y, Huang X, Grill R, Kim DH (2017). The systematic analysis of coding and long non-coding RNAs in the sub-chronic and chronic stages of spinal cord injury. Sci Rep.

[71] Zhou J, Fan Y, Chen H (2017). Analyses of long non-coding RNA and mRNA profiles in the spinal cord of rats using RNA sequencing during the progression of neuropathic pain in an SNI model. RNA Biol.

[72] Ray P, Torck A, Quigley L, Wangzhou A, Neiman M, Rao C (2018). Comparative transcriptome profiling of the human and mouse dorsal root ganglia: an RNA-seq-based resource for pain and sensory neuroscience research. Pain.

[73] Scott-Solomon E, Kuruvilla R (2018). Mechanisms of neurotrophin trafficking via Trk receptors. Mol Cell Neurosci.

[74] Wang GJ, Zhang X, Huang LD, Xiao Y. Involvement of the sodium channel Nav1.7 in paclitaxel-induced peripheral neuropathy through ERK1/2 signaling in rats. Curr Neurovasc Res. 2020;17(3):267–74. 10.2174/1567202617666200514113441 32407275

[75] Zhao YX, Yao MJ, Liu Q, Xin JJ, Gao JH, Yu XC (2020). Electroacupuncture treatment attenuates paclitaxel-induced neuropathic pain in rats via inhibiting spinal glia and the TLR4/NF-κB pathway. J Pain Res.

[76] Gelderblom H, Verweij J, Nooter K, Sparreboom A, Cremophor EL (2001). The drawbacks and advantages of vehicle selection for drug formulation. Eur J Cancer.

[77] Jia M, Wu C, Gao F, Xiang H, Sun N, Peng P (2017). Activation of NLRP3 inflammasome in peripheral nerve contributes to paclitaxel-induced neuropathic pain. Mol Pain.

[78] Manjavachi MN, Passos GF, Trevisan G, Araújo SB, Pontes JP, Fernandes ES (2019). Spinal blockage of CXCL1 and its receptor CXCR2 inhibits paclitaxel-induced peripheral neuropathy in mice. Neuropharmacology.

[79] Li D, Huang ZZ, Ling YZ, Wei JY, Cui Y, Zhang XZ (2015). Up-regulation of CX3CL1 via nuclear factor-kappaB-dependent histone acetylation is involved in paclitaxel-induced peripheral neuropathy. Anesthesiology.

