# Supplementary material for: Transcriptome profiling of long noncoding RNAs and mRNAs in spinal cord of a rat model of paclitaxel-induced peripheral neuropathy identifies potential mechanisms mediating neuroinflammation and pain
Source: J Neuroinflammation. 2021 Feb 18;18:48. doi: 10.1186/s12974-021-02098-y (PMC7890637; doi:10.1186/s12974-021-02098-y)
Supplement: Supplementary file 8 — Additional file 8. [file 12974_2021_2098_MOESM8_ESM.docx]

**Suppl Table13. The 10 DEGs of PINP overlapping with the SNI datasets**

| **Gene symbol** | **Official gene name (NCBI)** | **Change in three datasets** |
| --- | --- | --- |
| Cxcl13 | C-X-C motif chemokine ligand 13 | Up |
| Csf1r | colony stimulating factor 1 receptor | Up |
| Plac8 | placenta associated 8 | Up |
| Cd68 | Cd68 molecule | Up |
| Lgmn | legumain | Up |
| Ifngr1 | interferon gamma receptor 1 | Up |
| Timp1 | TIMP metallopeptidase inhibitor 1 | Up |
| Ggta1 | glycoprotein, alpha-galactosyltransferase 1 | Up |
| Clec4a3 | C-type lectin domain family 4, member A3 | Up |
| Grxcr1 | glutaredoxin and cysteine rich domain containing 1 | Up |

**Suppl Table14. The 10 DEGs of PINP overlapping with the CCI datasets**

| **Gene symbol** | **Official gene name (NCBI)** | **Change in three datasets** |
| --- | --- | --- |
| Cxcl13 | C-X-C motif chemokine ligand 13 | Up |
| Csf1r | colony stimulating factor 1 receptor | Up |
| Plac8 | placenta associated 8 | Up |
| Cd68 | Cd68 molecule | Up |
| Cyth4 | cytohesin 4 | Down |
| Gpr31 | G protein-coupled receptor 31 | Up |
| Cd22 | CD22 molecule | Up |
| Aoah | acyloxyacyl hydrolase | Up |
| Gapt | Grb2-binding adaptor protein, transmembrane | Up |
