# Supplementary material for: Transcriptome profiling of long noncoding RNAs and mRNAs in spinal cord of a rat model of paclitaxel-induced peripheral neuropathy identifies potential mechanisms mediating neuroinflammation and pain
Source: J Neuroinflammation. 2021 Feb 18;18:48. doi: 10.1186/s12974-021-02098-y (PMC7890637; doi:10.1186/s12974-021-02098-y)
Supplement: Supplementary file 9 — Additional file 9. [file 12974_2021_2098_MOESM9_ESM.docx]

**Suppl Table 15**

**The 4 DEGs of PINP overlapping with the SNI and CCI datasets**

| **Gene symbol** | **Official gene name (NCBI)** | **Change in three datasets** |
| --- | --- | --- |
| Cxcl13 | C-X-C motif chemokine ligand 13 | Up |
| Csf1r | colony stimulating factor 1 receptor | Up |
| Plac8 | placenta associated 8 | Up |
| Cd68 | Cd68 molecule | Up |
