# Supplementary figures and images for: Transcriptome profiling of long noncoding RNAs and mRNAs in spinal cord of a rat model of paclitaxel-induced peripheral neuropathy identifies potential mechanisms mediating neuroinflammation and pain
Source: J Neuroinflammation. 2021 Feb 18;18:48. doi: 10.1186/s12974-021-02098-y (PMC7890637; doi:10.1186/s12974-021-02098-y)

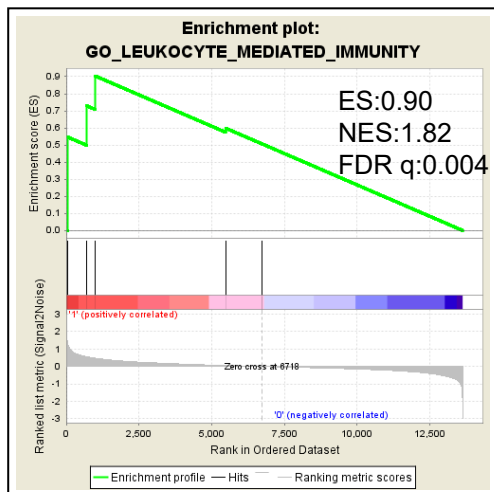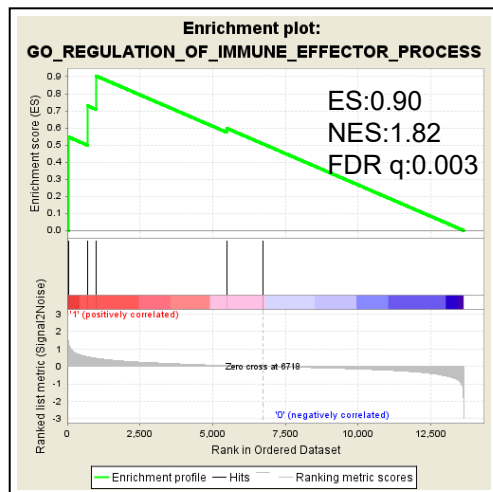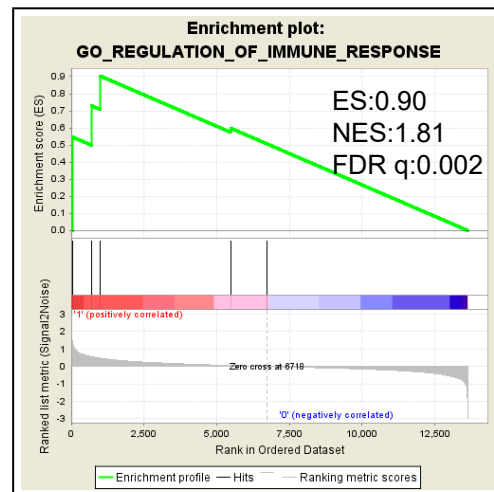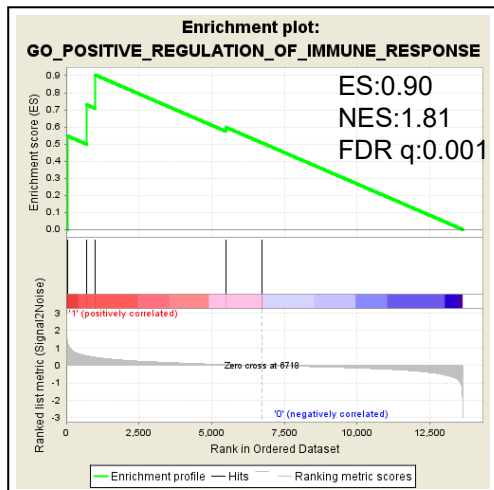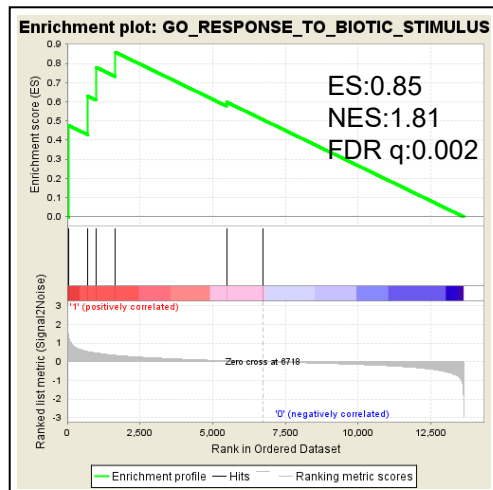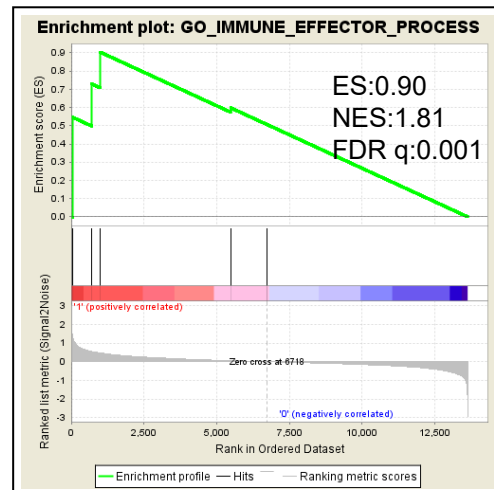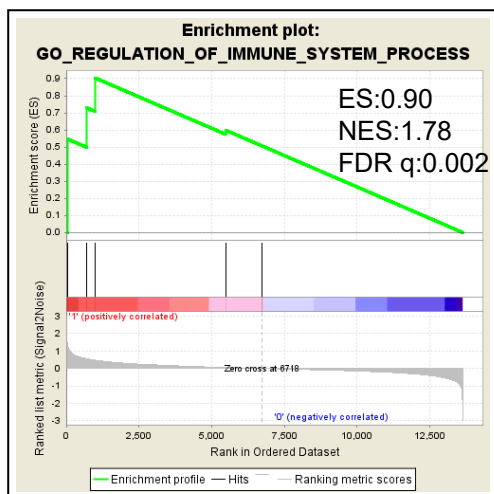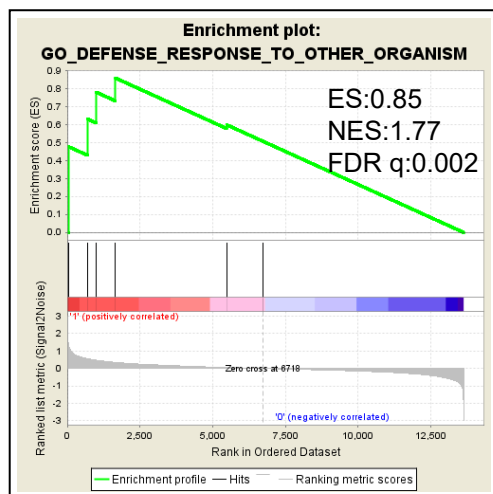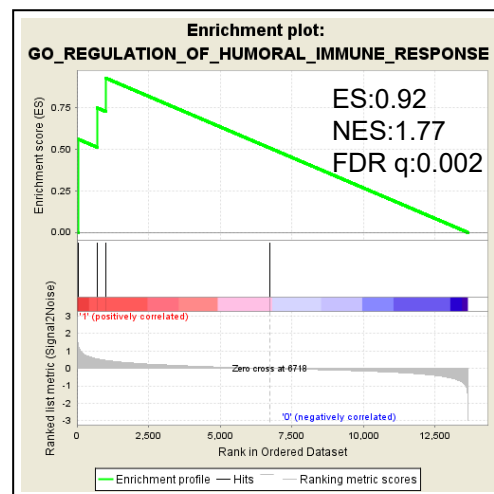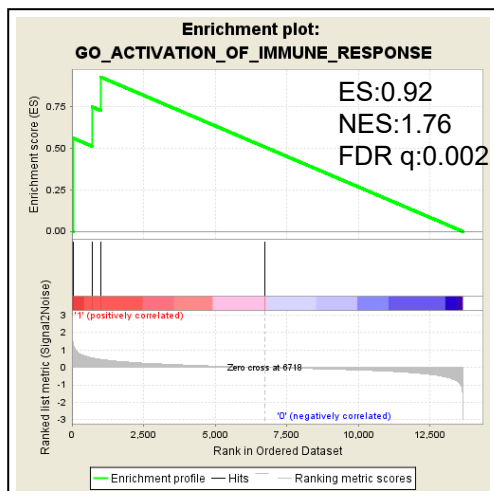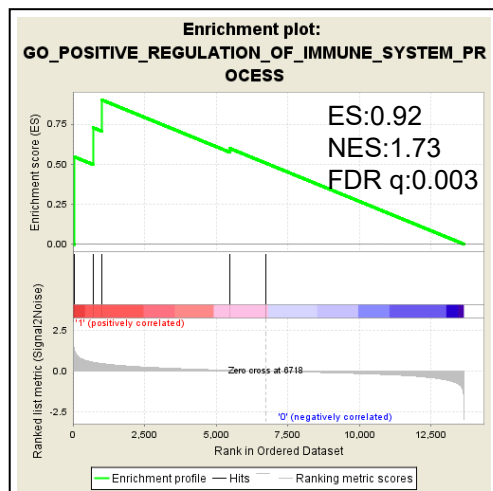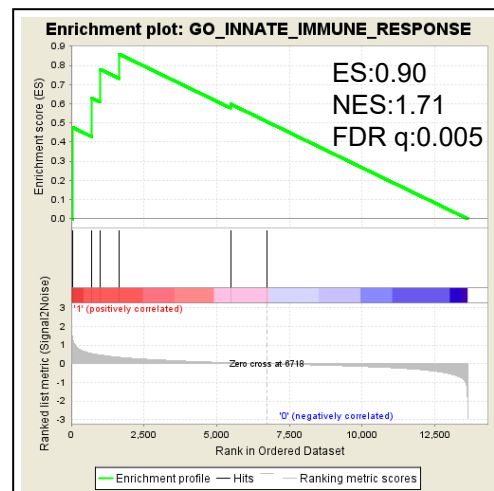

Supplement: Supplementary file 10 — Additional file 10: Suppl. Fig. 1. Gene Set Enrichment Analysis (GSEA) of enriched pathways from GO analysis. GSEA analysis of RNA-Seq dataset of PIPN model rats using well-defined gene sets derived from GO analysis. The top 12 most significantly upregulated pathways by paclitaxel treatment deduced from GSEA analysis was listed, with NES > 1.0 and FDR ≦ 0.25 as cut off threshold. [file 12974_2021_2098_MOESM10_ESM.pdf]

**A**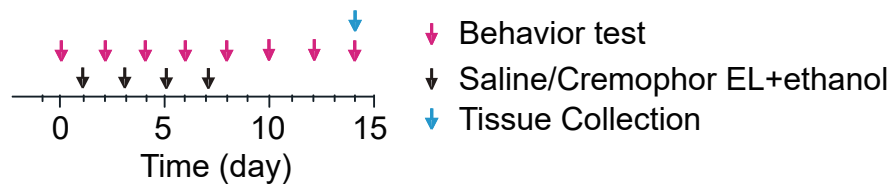**B**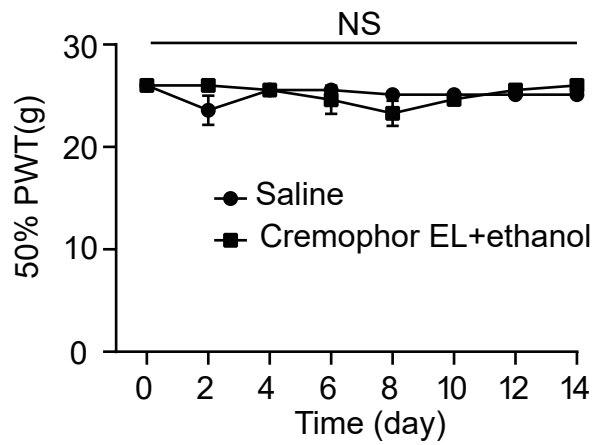**C**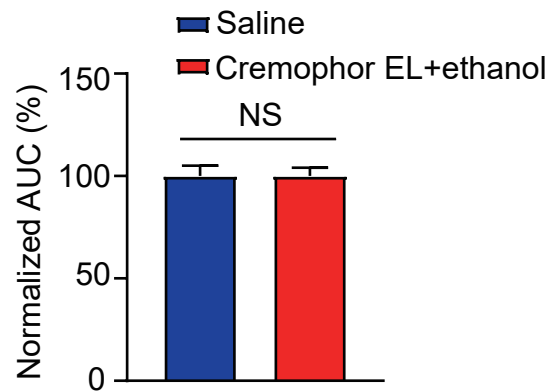**D**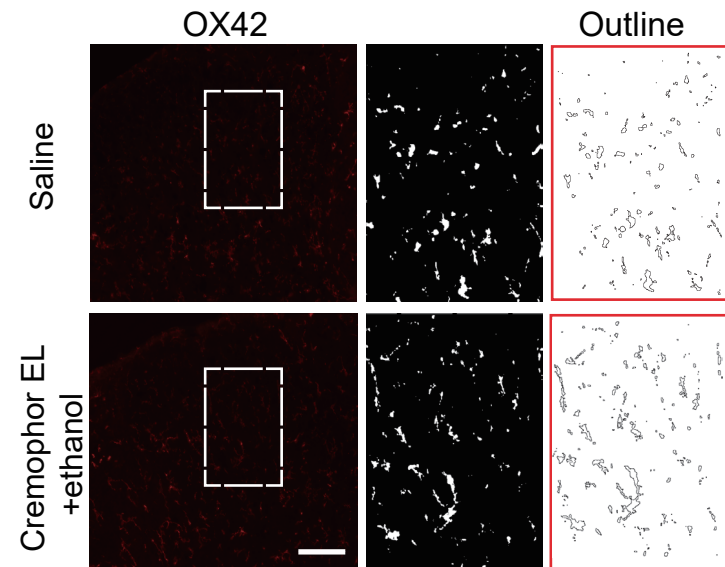**E**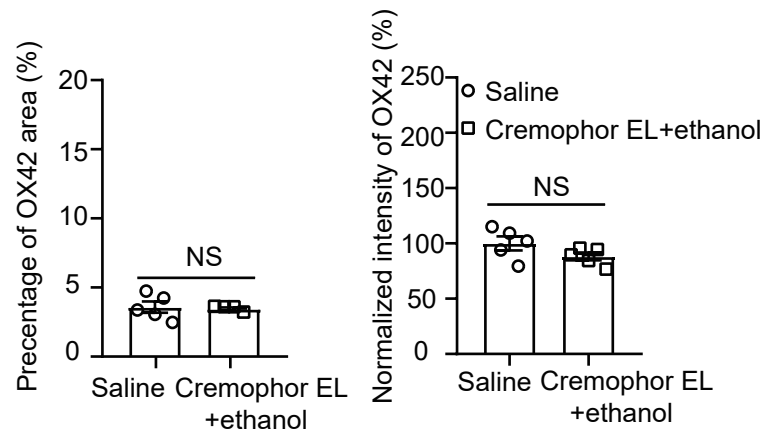**F**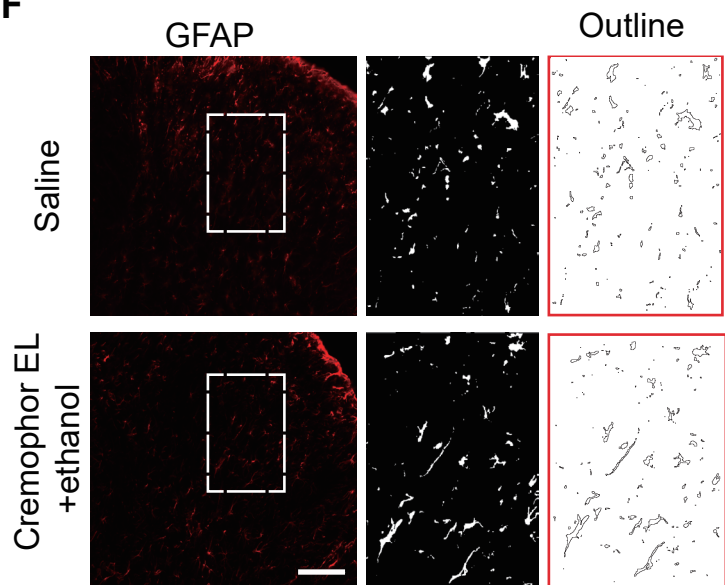**G**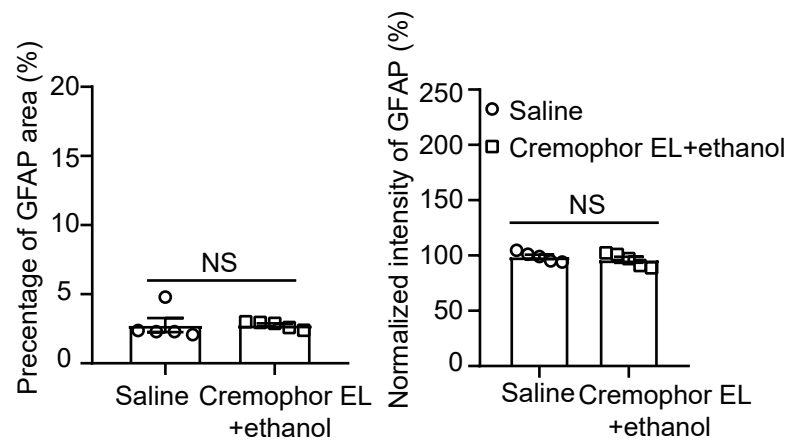

Supplement: Supplementary file 11 — Additional file 11: Suppl. Fig. 2. The comparison of the effects of 0.9% saline and Cremophor EL/ethanol on mechanical pain threshold and spinal glial activation in rats. (A) Experimental protocol for saline or Cremophor EL/ethanol treatment. Saline or Cremophor EL/ethanol (1:5 diluted in saline, according to dilution ratio of paclitaxel formulation used for PIPN model establishment) was injected (i.p.) with a volume of 0.5 ml/250 g body weight into rats at time points as indicated. (B) Effects of saline or Cremophor EL/ethanol on mechanical pain threshold. (C) Normalized AUC analysis of panel (A). (D) Representative immunofluorescence images indicating OX42 antibody staining of spinal cord from saline- and Cremophor EL/ethanol-treated groups. (E) Percentage of OX42 positively staining area in each observation field (left panel). Summary of the normalized % increase in fluorescence intensity of OX42 immunostaining in each observation field (right panel). The value of each group was normalized to that of saline group. (F) Representative immunofluorescence images indicating GFAP antibody staining of spinal cord from saline- and Cremophor EL/ethanol-treated groups. (G) Percentage of GFAP positively stained area in each observation field (left panel). Summary of the normalized % increase in fluorescence intensity of GFAP immunostaining in each observation field (right panel). The value of each group was normalized to that of saline group. n = 5 rats/group. NS: no significance. Student’s t test was used for statistical analysis. [file 12974_2021_2098_MOESM11_ESM.pdf]
